# Attenuated sensorimotor hemodynamic responses and altered cortical dynamics during passive proprioceptive-related stimulation after stroke: a multidimensional fNIRS study

**DOI:** 10.3389/fneur.2026.1848276

**Published:** 2026-09-16

**Authors:** Hui Sun, Qiuyuan Fu, Yichen Jiang, Guangyue Zhu

**Affiliations:** 1 School of Medicine, Tongji University, Shanghai, China; 2 Department of Neurology, Shanghai Tongji Hospital, School of Medicine, Tongji University, Shanghai, China; 3 School of Rehabilitation Science, Shanghai University of Traditional Chinese Medicine, Shanghai, China; 4 Department of Rehabilitation Medicine, Huadong Hospital, Fudan University, Shanghai, China

**Keywords:** cortical dynamic network, functional near-infrared spectroscopy, proprioceptive processing, recovery-phase complexity, stroke

## Abstract

**Objective:**

This study aimed to use functional near-infrared spectroscopy to investigate abnormalities in cortical activity and dynamic network mechanisms associated with proprioceptive processing after stroke. We integrated conventional hemodynamic features, latent-state organization, directed connectivity, and recovery-phase complexity analyses to explore multidimensional changes in cortical dynamics.

**Methods:**

Patients with stroke and healthy controls underwent baseline motor and sensory assessments and completed a block-designed proprioceptive task during fNIRS recording, consisting of six alternating 30-s stimulation and 30-s rest periods. After preprocessing, ROI-level oxygenated hemoglobin (HbO) time series were extracted. Conventional temporal features included area under the curve (AUC), onset latency, peak amplitude, post-stimulus residual activation, recovery slope, and time-to-peak. Latent state analysis was performed using a Gaussian mixture model to derive fractional occupancy (FO), mean dwell time (MDT), and transition characteristics. Directed connectivity during prestimulus, stimulation, and recovery phases was assessed using a sliding-window multivariate autoregressive model with partial directed coherence (PDC). Recovery-phase dynamics were further quantified using sample entropy, mean multiscale entropy, recurrence rate (RR), determinism (DET), and mean diagonal line length (Lmean).

**Results:**

A total of 62 participants, including 30 patients with stroke and 32 healthy controls, underwent a passive thumb movement task during fNIRS recording. Conventional HbO analysis showed that between-group differences were mainly confined to the left sensorimotor cortex, where the stroke group differed significantly from healthy controls in AUC (*p* = 0.023), peak amplitude (*p* = 0.003), and recovery slope (*p* = 0.018). No other ROI showed significant between-group differences, although marginal trends were observed in the right prefrontal and right sensorimotor cortices. Latent state analysis identified six states. Healthy controls more frequently occupied State 1 and State 5, whereas the stroke group more frequently occupied State 3 and State 6, and showed longer MDT in State 2 and State 3. Directed connectivity analysis revealed phase-dependent shifts in both groups, with the stroke group showing stronger prefrontal–sensorimotor reorganization across prestimulus, stimulation, and recovery phases. Recovery-phase complexity analysis demonstrated significantly higher global RR, DET, and Lmean in the stroke group, whereas sample entropy and mean multiscale entropy did not remain significant after correction. ROI-level complexity differences did not survive multiple-comparison correction.

**Conclusion:**

Abnormal proprioceptive processing after stroke is characterized not only by altered local activation, but also by dynamic state shifts, phase-specific reorganization of information flow, and increased regularity with reduced flexibility during recovery.

## 1 Background

Proprioception is a fundamental sensory basis for maintaining fine motor control of the upper limb. It continuously provides information about limb position, movement direction, movement amplitude, and the state of muscular effort. Unlike pure tactile sensation, proprioception relies more heavily on afferent signals from muscle spindles, Golgi tendon organs, joint receptors, and, to some extent, skin-stretch receptors, among which muscle spindles play a central role in detecting changes in muscle length and movement velocity (1). These afferent signals are used not only to determine the spatial position of the limb but also to support online motor correction, sensorimotor integration, and action prediction. Proprioceptive impairment can directly affect reaching, grasping, postural control, and fine manipulation of the upper limb and represents an important component of post-stroke motor dysfunction.

Upper-limb sensory impairment is common after stroke and may involve abnormalities in touch, discriminative sensation, vibration, pain and temperature sensation, as well as deficits in position sense and kinesthesia (2). Carey et al. reported that, among patients with stroke in the rehabilitation phase, approximately 47% had impaired tactile discrimination in the contralesional hand, approximately 49% had impaired position sense, and approximately 67% had impairment in at least one sensory modality (3). Previous studies have also suggested that proprioceptive impairment is associated with motor performance, length of hospital stay, quality of life, and rehabilitation outcomes (4). Therefore, post-stroke upper-limb dysfunction should not be understood solely in terms of insufficient motor cortical output; abnormalities in sensory input and their effects on sensorimotor circuits should also be considered. From a neurophysiological perspective, proprioceptive processing is not mediated by a single sensory cortical area but involves a distributed sensorimotor network comprising S1, S2, the posterior parietal cortex, premotor cortex (PMC), supplementary motor area (SMA), primary motor cortex (M1), and prefrontal regions. Previous fMRI studies have shown that passive proprioceptive input can induce longitudinal changes in sensorimotor cortical responses in patients with stroke and that these changes are related to the course of motor recovery (5). Studies of passive finger position sense have further suggested that post-stroke proprioceptive deficits cannot be explained solely by focal injury to sensory regions but require consideration of both structural damage within the sensorimotor system and its functional activity state (6). These findings indicate that proprioceptive-related abnormalities after stroke may manifest at multiple levels, including altered local activation strength, delayed cortical response timing, impaired interregional coordination, and disrupted post-task recovery.

In recent years, stroke recovery has increasingly been understood as a process of distributed brain network reorganization rather than dysfunction of a single lesion or brain region. Cassidy and Cramer emphasized that functional connectivity and brain network states may provide information about stroke recovery and treatment response beyond that captured by conventional clinical scales (7). Richards and Cramer likewise noted that a key objective of stroke rehabilitation is to promote beneficial neuroplasticity and network remodeling (8). Dynamic functional connectivity studies have further shown that stroke alters not only mean connectivity strength but also the occupancy, dwell time, and transition characteristics of different dynamic network states (9). A review by Wu et al. on static and dynamic functional connectivity after stroke also indicated that dynamic functional network analysis can complement conventional static approaches by revealing time-varying network reorganization associated with post-stroke motor dysfunction and recovery (10). Therefore, investigations of proprioceptive-related cortical activity after stroke should not be limited to average activation strength but should also examine temporal organization, state transitions, the direction of information flow, and recovery-phase dynamics.

Functional near-infrared spectroscopy can continuously record task-related changes in cortical oxygenated hemoglobin and is well suited for assessing dynamic hemodynamic responses during sensorimotor tasks in patients with stroke (11). Previous fNIRS studies have shown that upper-limb motor recovery after stroke is accompanied by dynamic reorganization of sensorimotor cortical hemodynamic patterns (12). However, most existing studies have focused on average task-related activation or static functional connectivity, with limited attention to the temporal structure of cortical responses during passive proprioceptive stimulation, full-time activity states, phase-specific directed connectivity, and recovery-phase complexity. In patients with stroke, proprioceptive-related cortical abnormalities may not be limited to reduced peak responses but may also involve delayed response onset, slower post-stimulus HbO return, abnormal persistence of cortical activity states, reorganization of interregional information flow, and reduced signal flexibility during recovery.

Against this background, the present study used functional near-infrared spectroscopy to record cortical HbO responses evoked by passive thumb movement in patients with stroke and healthy controls and established a multidimensional dynamic analysis framework. Conventional HbO temporal features were used to quantify the amplitude, peak, onset timing, and post-stimulus hemodynamic recovery of task-evoked responses. Full-time latent-state analysis was used to identify recurrent cortical activity states during the task and characterize their occupancy and dwell properties. Directed connectivity analysis was used to describe phase-specific information-flow patterns among brain regions during the pre-stimulation, stimulation, and recovery phases. Recovery-phase complexity analysis was used to evaluate whether post-stimulus HbO signals exhibited increased regularity, reduced flexibility, or abnormal nonlinear dynamics. By integrating these complementary measures, this study aimed to characterize cortical dynamic abnormalities associated with passive proprioceptive stimulation after stroke across four levels—local activation, dynamic states, directed networks, and recovery-phase complexity—and to provide a more systematic body of fNIRS evidence for understanding post-stroke sensorimotor circuit dysfunction.

## 2 Methods

### 2.1 Participants

All participants in this study provided written informed consent prior to enrollment. The inclusion criteria for the stroke group were as follows: meeting the diagnostic criteria of the Chinese Guidelines for the Diagnosis and Treatment of Acute Ischemic Stroke (2018) or the Chinese Guidelines for the Diagnosis and Treatment of Intracerebral Hemorrhage (2019); first-ever stroke with a unilateral lesion, or a previous history of stroke without obvious residual neurological dysfunction; age between 25 and 85 years; disease duration longer than 1 month; a Mini-Mental State Examination (MMSE) score >21; and provision of written informed consent by both the patient and family members. The exclusion criteria were as follows: a personal or family history of epilepsy; marked sensory loss or hypersensitivity to stimulation; and severe comorbid internal diseases, such as significant hepatic or renal dysfunction. Healthy controls were all right-handed, had no history of neurological, physical, psychological, or psychiatric disorders, were able to understand the study procedures, and provided written informed consent. The study protocol was approved by the Ethics Committee of Yueyang Hospital of Integrated Traditional Chinese and Western Medicine affiliated with Shanghai University of Traditional Chinese Medicine (2023–132) and was registered with the Chinese Clinical Trial Registry (ChiCTR2300072924).

### 2.2 Functional assessment

All participants underwent baseline data collection and functional assessment before fNIRS acquisition. The assessments were conducted in a quiet environment by uniformly trained researchers and were primarily used to characterize participants’ demographic features, the side tested during the experiment, upper-limb motor function in patients with stroke, and proprioceptive status. Age, sex, and the hand tested during the experiment were recorded for all participants. In the healthy control group, the tested side was recorded as the right or left hand according to the hand used to complete the task. In the stroke group, the tested side was recorded as the left or right side according to the side assessed. These data were used to describe the basic characteristics of the two groups and to compare their comparability in terms of age, sex, and tested-side distribution. Upper-limb motor function in patients with stroke was assessed using the Fugl–Meyer Assessment of the Upper Extremity. This scale is used to quantify the severity of upper-limb motor impairment after stroke, with higher scores indicating better upper-limb motor function. The total FMA-UE score was recorded for the stroke group to characterize baseline motor function. Position sense was assessed separately on the left and right sides. During the assessment, participants kept their eyes closed while the examiner passively moved the thumb or finger joints and asked them to identify the direction of joint movement or the final joint position. Left- and right-side position-sense scores were recorded separately for patients with stroke to characterize bilateral proprioceptive status. Position sense was rated using the predefined 0–3 clinical grading scale in the case report form, with higher scores indicating more severe impairment. This measure was primarily used to describe baseline sensory status in patients with stroke and to assist in interpreting differences in cortical activity evoked by the passive thumb movement task.

### 2.3 Devices and paradigm

Cortical hemodynamic signals were acquired using a functional near-infrared spectroscopy (fNIRS) brain imaging system (BS-2000 L; Wuhan Zilian Hongkang Technology Co., Ltd., Wuhan, China). The system was equipped with dual-wavelength light sources at 690 and 830 nm and operated at a sampling frequency of 10 Hz. The optode array consisted of 27 sources and 25 detectors mounted on a flexible cap, with an inter-optode distance of 3 cm. The optode configuration was arranged according to the international 10–20 system, with S2 positioned at Fpz. A three-dimensional digitizer (NirMap; Wuhan Zilian Technology Co., Ltd., Wuhan, China) was used to record the spatial coordinates of the nasion (Nz), vertex (Cz), left preauricular point (AL), right preauricular point (RL), and all optodes. Channels were then projected onto the cortical surface and mapped to Brodmann areas using the NIRS-SPM approach. In accordance with the study objectives, the primary regions of interest (ROIs) included the bilateral prefrontal cortex and sensorimotor cortex.

This study used a two-level ROI definition. To analyze the overall task-evoked hemodynamic response, channel-level signals were combined into four macroscopic ROIs: the left prefrontal cortex, left sensorimotor-related cortex, right prefrontal cortex, and right sensorimotor-related cortex. This four-ROI grouping was primarily used for block-averaged HbO/HbR curves and conventional temporal-feature analyses to improve the stability of regional mean signals and reduce the burden of multiple comparisons. To further investigate the dynamic organization of brain activity states, directed information flow, and recovery-phase complexity, 17 subdivided ROIs were defined, including the bilateral secondary somatosensory cortices, somatosensory-related cortices, motor-related cortices, premotor cortices, supplementary motor areas, ventromedial prefrontal cortices, dorsolateral prefrontal cortices, dorsomedial prefrontal cortices, and the frontal pole region. Compared with the four macroscopic ROIs, the 17 subdivided ROIs better preserved spatial differences within the sensorimotor and prefrontal networks and were therefore used for latent-state analysis, PDC-based directed connectivity analysis, and recovery-phase complexity analysis. The anatomical assignment of ROIs was determined based on the spatial localization of the fNIRS channels. A three-dimensional digitization system was used to record the nasion, Cz, bilateral preauricular points, and the positions of all sources and detectors. Channel locations were then projected into standard brain space using the NIRS-SPM method. Each channel was assigned to the corresponding anatomical or functional region according to its projected location and associated Brodmann area or cortical label. Adjacent channels belonging to the same major functional region were combined into the corresponding ROI. The specific channel composition of each ROI is provided in the Supplementary Datasheets 1–3.

A manually standardized passive thumb movement task was used as a sensory stimulation paradigm primarily involving proprioceptive input. The thumb was selected as the stimulation site for the following reasons. The thumb and hand joints are closely related to fine motor control of the upper limb and are clinically relevant sites of post-stroke upper-limb dysfunction. Passive joint movement can elicit sensory afferent input related to joint position sense and kinesthesia while minimizing the influence of active motor execution and motor intention (33). The task is simple to administer, well tolerated, and suitable for fNIRS recording in patients with stroke while seated in a natural position.

To improve task standardization, all stimulation was administered by therapists who had received uniform training. Before the experiment, participants were seated comfortably with both upper limbs resting naturally on their thighs, and the tested hand was kept relaxed. Before formal data acquisition, the researchers provided standardized instructions and conducted a brief practice session to ensure that participants understood the task requirements. At the beginning of each stimulation period, participants closed their eyes while the therapist gently held the distal part of the thumb, taking care to avoid excessive skin pressure and friction, and passively moved the thumb at a frequency of approximately 1 Hz. The movements included flexion, extension, and small positional changes in different directions. Participants were instructed to perceive and remember the position of the thumb as accurately as possible without actively moving or providing verbal responses. Each stimulation block lasted 30 s, followed by 30 s of rest, and a total of six blocks were performed.

It should be noted that manual passive thumb movement inevitably involves some degree of skin contact and tactile input. Therefore, this paradigm cannot completely isolate proprioceptive input from tactile input. To minimize non-target tactile stimulation, the therapist used a light grip to stabilize the distal thumb and avoided obvious skin compression and friction during stimulation. Participants kept their eyes closed throughout the task to eliminate visual input and were instructed not to move actively or respond verbally, thereby reducing brain activity related to motor execution and language responses. Accordingly, this task is more accurately understood as a passive sensory stimulation paradigm dominated by joint position and kinesthetic input, while potentially containing a small tactile component.

### 2.4 fNIRS data preprocessing

The sensorimotor cortex on the lesioned side in the stroke group was uniformly flipped to the left hemisphere, whereas the remaining cortical regions were not flipped (Supplementary Datasheet 4). fNIRS data preprocessing was performed using the Homer2 platform. Raw light intensity signals were first converted into optical density (OD) signals. Stimulus-related artifacts were identified and removed using the enStimRejection procedure, with the artifact-rejection window set from 5 s before stimulus onset to 10 s after stimulus onset. Subsequently, the OD signals were band-pass filtered with a high-pass cutoff of 0.01 Hz and a low-pass cutoff of 0.10 Hz to remove low-frequency drift and high-frequency physiological noise. The filtered OD signals were then converted into hemoglobin concentration changes according to the modified Beer–Lambert law, with the partial pathlength factor (PPF) set to 6.0 for both wavelengths. Principal component analysis-based denoising was not further applied in the present study. Finally, task-related hemodynamic responses were extracted using a block-averaging approach, with the analysis window defined from 2 s before stimulus onset to 60 s after stimulus onset (11, 13, 14). Because HbO provides a higher signal-to-noise ratio and is more sensitive to task-evoked hemodynamic changes, subsequent statistical analyses were primarily based on HbO signals. After preprocessing, HbO signals from all channels within each predefined ROI were averaged across the temporal dimension to obtain ROI-level time series. Different analysis modules incorporated additional signal-processing procedures according to their specific analytical purposes. For the block-averaged temporal feature analysis, no z-score normalization was applied before feature extraction. ROI-level HbO/HbR curves were obtained by averaging the block-averaged signals output by Homer2 across channels within each ROI. Temporal features were calculated relative to the pre-stimulus baseline, thereby preserving between-group differences in amplitude-related measures, including AUC, peak amplitude, and recovery slope. For the latent-state analysis, each participant’s ROI-level HbO time series was z-score normalized before construction of the shared state space. For the PDC-based directed connectivity analysis, ROI time series were detrended and z-score normalized before multivariate autoregressive modeling. For the recovery-phase complexity analysis, recovery-phase signals were detrended and baseline-corrected before entropy and recurrence analyses. The normalization formula was defined as follows:


$$z_{r}(t)=\frac{x_{r}(t)-\mu_{r}}{\sigma_{r}}$$

Here, x_r(t) represents the raw HbO signal of the r-th ROI at time t, while \mu_r and \sigma_r denote the mean and standard deviation of the ROI’s time series, respectively (Figure 1).

**Figure 1:**
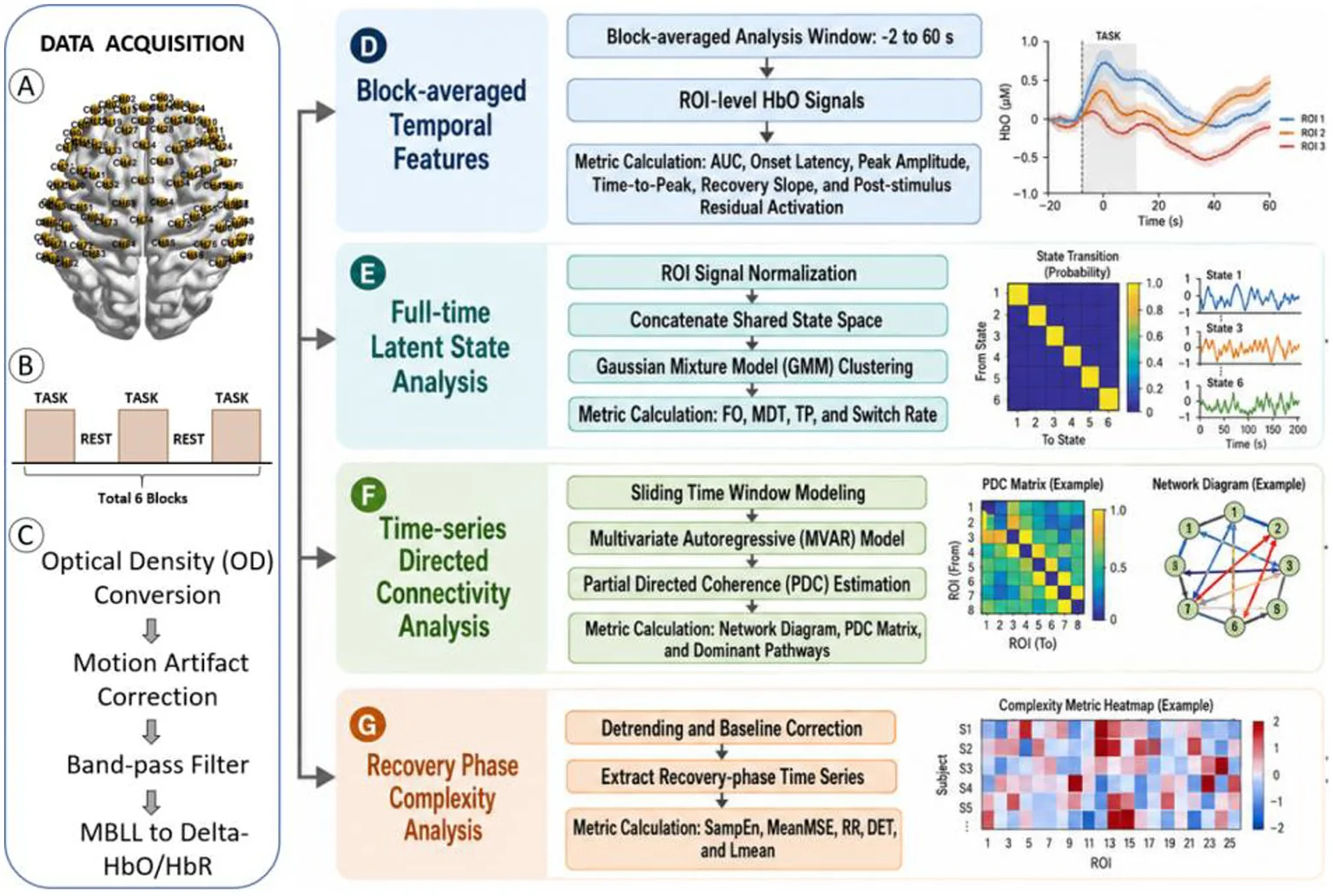
Overview of the fNIRS acquisition and multidimensional analysis workflow. **(A)** fNIRS channel configuration. **(B)** Passive thumb movement paradigm with alternating task and rest blocks. **(C)** Preprocessing pipeline, including optical density conversion, motion artifact correction, band-pass filtering, and modified Beer–Lambert law conversion to HbO/HbR signals. **(D)** Block-averaged temporal feature analysis of ROI-level HbO signals. **(E)** Gaussian mixture model-based latent state analysis, including fractional occupancy (FO), mean dwell time (MDT), transition probability (TP), and switch rate. **(F)** Sliding-window multivariate autoregressive modeling and partial directed coherence (PDC) analysis of directed connectivity. **(G)** Recovery-phase complexity analysis using sample entropy (SampEn), mean multiscale entropy (MeanMSE), recurrence rate (RR), determinism (DET), and mean diagonal line length (Lmean).

### 2.5 Block-averaged temporal feature analysis

To characterize the task-related hemodynamic response, temporal feature analysis was performed on the block-averaged HbO curves. Using the mean and standard deviation of the prestimulus baseline window as reference values, key parameters during both the activation and recovery phases were calculated. For the block-averaged temporal feature analysis, the interval from −5 to 0 s before stimulus onset was defined as the baseline window and was used to calculate the baseline mean and standard deviation for each ROI. The primary task-response window was defined as 1–30 s after stimulus onset. Onset latency was defined as the first time point after stimulus onset at which the HbO signal continuously exceeded the baseline mean + *κ* × baseline SD, with κ set to 2 and at least two consecutive sampling points required to exceed the threshold. Peak amplitude and time-to-peak were calculated within the task-response window. The recovery-phase endpoint was defined as 45 s after stimulus onset, corresponding to 15 s after the end of the 30-s stimulation period. Recovery slope was obtained by linearly fitting the HbO signal from the peak to the recovery-phase endpoint. Post-stimulus residual activation was defined as the mean HbO deviation from baseline during the late recovery window from 30 to 45 s after stimulus onset.

The baseline mean was defined as


$$\mu_{\text{base}}=\frac{1}{N_{\text{base}}}\sum_{t\in T_{\text{base}}}y(t)$$

The baseline standard deviation was defined as,


$$\sigma_{\text{base}}=\sqrt{\frac{1}{N_{\text{base}}-1}\sum_{t\in T_{\text{base}}}{\left(y(t)-\mu_{\text{base}}\right)}^{2}}$$

y(t) represents the ROI-level HbO signal, $T_{\text{base}}$ is the baseline time window.

### 2.6 Whole-time-course latent state analysis

To characterize the dynamic organization of brain activity patterns during task performance, latent state analysis was performed on the ROI-level HbO time series. First, the time series of each ROI for each participant were standardized. Subsequently, ROI signals from all participants and all time points were concatenated to construct a shared state space, and clustering was performed using a Gaussian mixture model (GMM). Each participant’s ROI-level HbO time series was z-score normalized before state modeling to reduce interindividual differences in signal amplitude. The standardized ROI time series from all participants and all time points were then concatenated to construct a shared state space. The candidate number of states was set from K = 2 to K = 6. For each candidate K value, GMM fitting was performed with 20 replicate initializations, a diagonal covariance matrix, and a regularization parameter of 1 × 10^−5^ to reduce the risk of covariance matrix singularity. A fixed random seed was used to ensure reproducibility. Let the observation vector be $x\in {\mathbb{R}}^{p}$ , where 𝑝 denotes the number of ROIs; the GMM can then be expressed as


$$p(x)=\sum_{k=1}^{K}\pi_{k}\;N(x|\mu_{k},\Sigma_{k})$$

Here, K denotes the number of latent states, πk represents the mixing weight for the k-th state, and μk and Σk correspond to the mean vector and covariance matrix for that state, respectively. Model selection was performed by comparing the AIC and BIC values of candidate models with *K* = 2–6 states. Model selection was performed by comparing the AIC and BIC values of candidate models with *K* = 2–6 states. Because BIC continued to decrease across the prespecified candidate range without reaching a clear internal minimum, the six-state solution was retained as a prespecified and interpretable main-analysis model rather than as a definitive BIC-optimal solution. Each time point was then assigned to a specific state according to the maximum posterior probability principle.


AIC=2q−2lnL,BIC=qlnn−2lnL

Where 𝑞 denotes the number of model parameters, 𝐿 is the likelihood function of the model, and 𝑛 is the sample size. The number of states corresponding to the minimum Bayesian information criterion (BIC) was selected as the model. Each time point was then assigned to a specific state according to the maximum posterior probability principle. Based on these state assignments, fractional occupancy (FO), mean dwell time (MDT), switch rate, and transition probability (TP) were further derived. FO reflects the proportion of all valid time points occupied by a given state; MDT quantifies the stability of a state in terms of its continuous persistence; switch rate characterizes the frequency of state transitions per unit time; and TP describes the transition pathways and their associated probabilities between different latent states. Because the GMM was fitted using the complete task time series rather than stimulation-period data alone, the identified latent states should be interpreted as recurrent cortical activity patterns occurring throughout the entire passive proprioceptive stimulation task, rather than simply as proprioceptive states specific to the stimulation period. State metrics were subsequently calculated within the predefined stimulation and recovery phases to further characterize the phase-specific properties of these states.

### 2.7 Time-series directed connectivity analysis

To evaluate the directional flow of information between brain regions, directed connectivity analysis was performed on the continuous ROI-level HbO time series. First, the signals from each ROI were detrended and standardized using z-scores. A sliding-window approach was then applied to model the dynamic connectivity process in a temporally segmented manner, with a window length of 20 s and a step size of 1 s, in order to characterize time-varying connectivity patterns across the prestimulus, stimulation, and recovery phases. For each time window, a multivariate autoregressive (MVAR) model was fitted to the multivariate signals.


$$X(t)=\sum_{k=1}^{p}A_{k}X(t-k)+E(t)$$

Here, X(t) ∈ ℝᴺ denotes the ROI observation vector at time t, where N is the number of ROIs, p is the model order, A_k_ is the regression coefficient matrix of order k, and E(t) is the residual term. The model order is automatically selected from a candidate range based on the AIC and BIC criteria, with the order corresponding to the minimum BIC value designated as the model.

Subsequently, the MVAR model is transformed into the frequency domain to construct the frequency-domain system matrix.


$$A(f)=I-\sum_{k=1}^{p}A_{k}e^{-i2\mathrm{\pi fk}/f_{s}}$$

Here, I denotes the identity matrix, and fs represents the sampling frequency. Furthermore, based on Partial Directed Coherence (PDC), the directional information flow between ROIs is estimated; the PDC directed from ROI j to ROI i is defined as


$$\pi_{\mathrm{ij}}(f)=\frac{A_{\mathrm{ij}}(f)}{\sqrt{\sum_{m=1}^{N}{|A_{\mathrm{mj}}(f)|}^{2}}}$$

Given that fNIRS signals are predominantly characterized by low-frequency oscillations, this study averaged the PDC values within the 0.01–0.08 Hz frequency band to derive the mean directional connectivity strength. Based on the center time point of each sliding window, all time windows were categorized into pre-task, stimulation, and recovery phases; subsequently, the mean PDC matrix was computed for each respective phase. Furthermore, the input and output strengths of each brain region were extracted to reflect, respectively, that region’s network role as an information source and as an information sink.

### 2.8 Recovery-phase complexity analysis

To assess the characteristics of dynamic complexity during the recovery of brain activity following the completion of a task, a complexity analysis was performed on region-of-interest (ROI) level HbO time series during the recovery phase. First, the signals underwent linear detrending and baseline correction using the mean of the pre-stimulus time window; subsequently, the time window corresponding to the recovery phase immediately following the task’s conclusion was extracted for analysis. The recovery-phase complexity analysis window was defined as the 25-s interval immediately following the end of the 30-s stimulation period. Because the extracted time series included a 10-s pre-stimulus baseline and the stimulation period was encoded as 10–40 s on the analysis timeline, the recovery window was set to 40–65 s, corresponding to 30–55 s after stimulus onset. This recovery-phase segment was used to calculate SampEn, MeanMSE, and recurrence quantification analysis metrics (15–17). Based on this recovery-phase time series, several metrics were computed: Sample Entropy (SampEn), Mean Multiscale Entropy (MeanMSE), and Recurrence Quantification Analysis (RQA) indices—specifically, Recurrence Rate (RR), Determinism (DET), and Mean Diagonal Line Length (Lmean). Multiscale entropy analysis was performed across scales 1–5, with a requirement that at least 20 data points remained after coarse-graining at each scale. The recurrence quantification analysis parameters were set as follows: embedding dimension = 2, delay = 1, recurrence radius = 0.1 × the standard deviation of the recovery-phase signal, and minimum diagonal line length = 2. Based on these parameters, recurrence rate, determinism, and mean diagonal line length were calculated.

The parameters for Sample Entropy are set to an embedding dimension of m = 2 and a similarity tolerance of r = 0.2 × SD. For a time series x = {x (1), x (2), …, x(N)}, template vectors of length m are constructed as Xi(m) = [x(i), x(i + 1), …, x(i + m − 1)]; if the Chebyshev distance between two such templates is less than the threshold r, they are recorded as a match. Sample Entropy is defined as:


$$\text{SampEn}(m,r,N)=-\ln(\frac{A}{B})$$

Here, B represents the number of m-dimensional matches, while A represents the number of (m + 1)-dimensional matches. A higher SampEn value indicates that the brain signals during the recovery phase are more irregular and exhibit greater complexity. Multiscale Entropy analysis involves coarse-graining the original time series at various scales, calculating the Sample Entropy for each respective scale, and ultimately computing the mean of these values to reflect the overall complexity across multiple time scales. In Recurrence Quantification Analysis (RQA), a distance matrix between state points is calculated via phase space reconstruction to construct a recurrence matrix; subsequently, the metrics RR, DET, and Lmean are computed to reflect, respectively, the recurrence of system states, the determinism of the dynamical structure, and the average duration of similar evolutionary trajectories. Collectively, the aforementioned metrics provide a comprehensive characterization of the brain activity recovery process from the perspectives of irregularity, multiscale complexity, and dynamical stability during the recovery phase.

### 2.9 Statistical analysis

Statistical analyses were performed using MATLAB. Before between-group comparisons, continuous variables were assessed for distributional characteristics and homogeneity of variance. Depending on the data distribution and variance assumptions, independent-samples t tests, Welch’s t tests, or Mann–Whitney U tests were used. Categorical variables were analyzed using the χ^2^ test or Fisher’s exact test. The main statistical analyses included ROI-level HbO temporal features, latent-state metrics, phase-specific ROI-to-ROI directed connectivity, and recovery-phase complexity metrics. Because return-to-baseline time could not be estimated reliably in some brain regions, it was not included in formal statistical inference. For analyses involving multiple comparisons, the Benjamini–Hochberg false discovery rate procedure was applied within each predefined family of analyses. Dominant pathways in the directed connectivity analysis were used only to describe dynamic changes in the strongest information-flow patterns across task phases and were not treated as independent inferential statistical results. All tests were two-sided. For analyses not involving multiple comparisons, statistical significance was defined as *p* < 0.05. For analyses involving multiple comparisons, statistical significance was defined as an FDR-adjusted q < 0.05. Detailed statistical procedures and parameter settings for each analysis module are provided in Supplementary Datasheet 5. This article contains numerous statistical results. For results not presented in the main text, please refer to Supplementary Datasheet 6.

## 3 Results

### 3.1 Demographic characteristics and functional assessment results

The baseline demographic and clinical characteristics are summarized in Table 1. The healthy control and stroke groups did not differ significantly in age, sex distribution, or tested hand/affected side distribution. For patients with stroke, the median disease duration was 6.33 months [IQR, 3.25–24.33], and the mean FMA-UE score was 32.50 ± 22.44. Left and right position sense scores are also reported in Table 1.

**Table 1:** Baseline demographic and clinical characteristics.

Variable	Healthy controls (*n* = 32)	Patients with stroke (*n* = 30)	*p*-value
Age, years	56.59 ± 8.70	61.27 ± 10.60	0.080
Sex, male/female	13/19	19/11	0.074
Tested hand / affected side, R/L	20/12	14/16	0.316
Disease duration, months	NA	6.33 [3.25, 24.33]	NA
FMA-UE	NA	32.50 ± 22.44	NA
Position sense score, left	NA	0.00 [0.00, 1.00]	NA
Position sense score, right	NA	0.00 [0.00, 1.00]	NA
Left position sense score distribution, 0/1/2/3	NA	19/4/5/2	NA
Right position sense score distribution, 0/1/2/3	NA	21/8/1/0	NA

Values are presented as mean ± SD, median [IQR], or counts, as appropriate. Age was compared using the Wilcoxon rank-sum test. Sex and tested hand/affected side were compared using the chi-square test. Disease duration, FMA-UE, and position sense scores were available only for patients with stroke; therefore, between-group comparisons were not performed for these variables. FMA-UE, Fugl-Meyer Assessment of the Upper Extremity; NA, not applicable.

### 3.2 Stroke patients show attenuated sensorimotor HbO responses and delayed post-stimulus hemodynamic recovery

Figure 2A presents the group-averaged HbO and HbR time courses across the four macro-ROIs. The curves were used to visualize the overall temporal profiles, whereas formal between-group interpretations were based on the predefined and statistically tested temporal features. In the LPFC and RPFC regions, the stroke group demonstrated relatively more pronounced HbO fluctuations during the stimulation phase; conversely, in the bilateral sensorimotor regions—particularly the RSMC—the stroke group exhibited a broader and more prolonged trend of negative HbO deviation during the post-stimulation phase. However, these morphological differences at the curve level did not translate into statistically significant differences across all extracted parameters. For the AUC metric (Figure 2B), the stroke group showed a lower value than the healthy group in the LSMC (*p* = 0.023; FDR-adjusted *q* = 0.043), indicating an attenuated cumulative task-evoked HbO response. Similarly, peak amplitude in the LSMC was lower in the stroke group than in the healthy group (Figure 2D, *P* = 0.003; FDR-adjusted *q* = 0.017), indicating a reduced task-evoked hemodynamic response magnitude. The recovery slope in the LSMC was less negative and closer to zero in the stroke group (Figure 2F, *P* = 0.018; FDR-adjusted *q* = 0.043), reflecting a flatter post-peak decline and slower hemodynamic recovery. No significant between-group differences in these metrics were observed in the other three ROIs (all *p* > 0.05). For the remaining temporal features—including onset latency (Figure 2C), time-to-peak (Figure 2G), and post-stimulus residual activation (Figure 2E)—no statistically significant differences were found between the two groups across any of the ROIs (all *p* > 0.05). Specifically, the time-to-peak in the RPFC exhibited a nearly significant trend (*p* = 0.077; FDR-adjusted *q* = 0.602), and the recovery slope in the RSMC also showed a marginal difference (*p* = 0.051; FDR-adjusted *q* = 0.327); however, neither reached the threshold for statistical significance. The return-to-baseline time was not included in further statistical interpretation, as it could not be reliably estimated across multiple ROIs in both groups.

**Figure 2:**
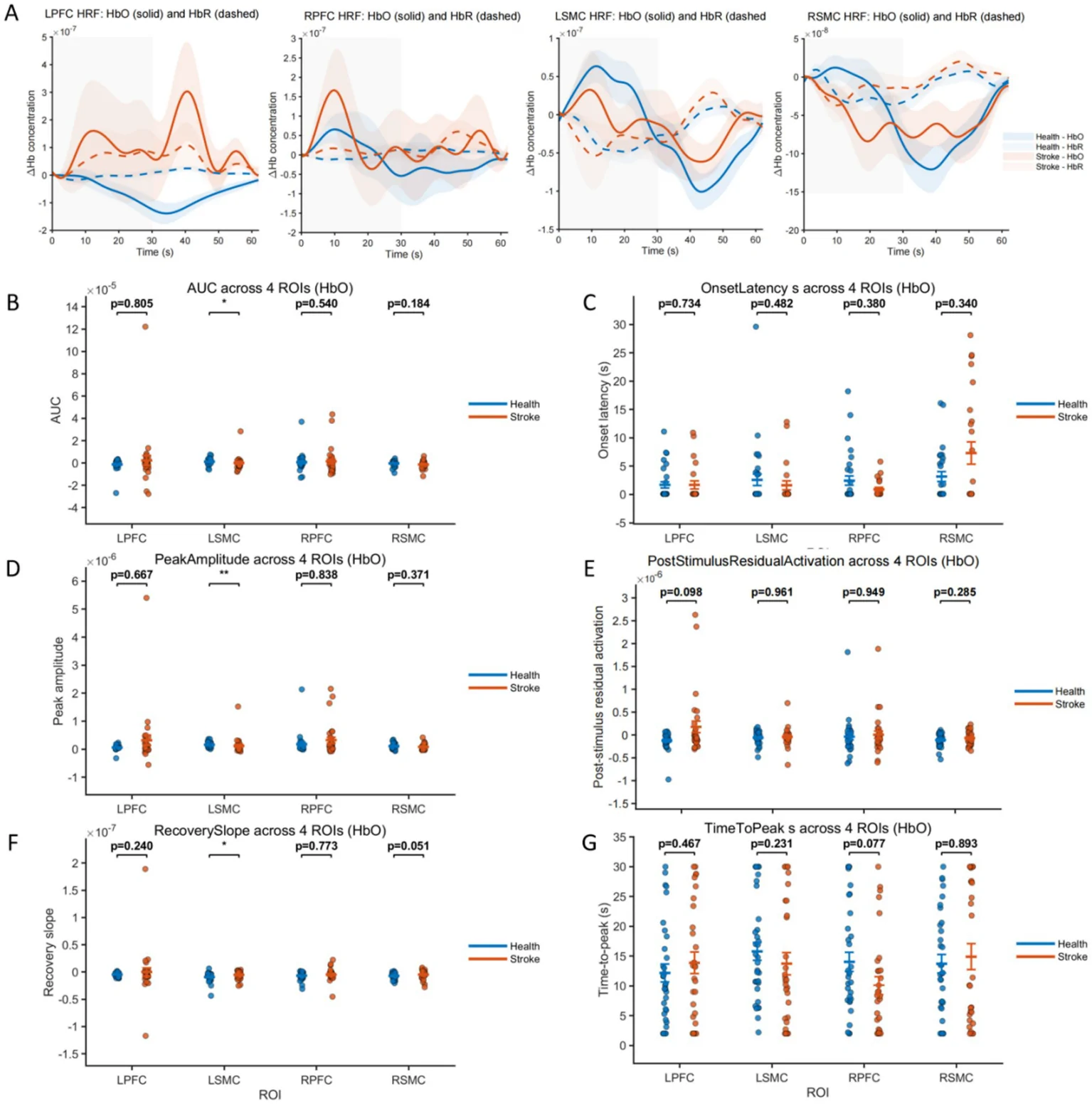
Comparison of average hemodynamic response curves and HbO temporal features across four ROIs between the healthy group and the stroke group. **(A)** Group-averaged hemodynamic response functions (HRFs) for the left prefrontal cortex (LPFC), left sensorimotor cortex (LSMC), right prefrontal cortex (RPFC), and right sensorimotor cortex (RSMC). Solid lines represent HbO, dashed lines represent HbR, and shaded regions indicate the standard error of the mean (SEM). Blue lines represent the healthy group, and orange lines represent the stroke group. **(B–G)** Comparison of HbO-derived temporal features for each ROI, including area under the curve (AUC), onset latency, peak amplitude, post-stimulus residual activation, recovery slope, and time-to-peak. Each data point represents a single subject, and error bars indicate the mean ± SEM. The *p*-values labeled above each ROI represent the uncorrected results of the corresponding group comparisons. LPFC, left prefrontal cortex; LSMC, left sensorimotor cortex; RPFC, right prefrontal cortex; RSMC, right sensorimotor cortex; HbO, oxyhemoglobin; HbR, deoxyhemoglobin; HRF, hemodynamic response function; SEM, standard error of the mean.

### 3.3 Stroke patients exhibit altered latent-state occupancy and prolonged dwell time

Figure 3 presents the results of the latent state analysis—constructed based on ROI-level HbO signals—for both the healthy control group and the stroke group. These results encompass state-specific spatial patterns, representative state sequences, fractional occupancy, mean dwell time, and state transition characteristics. Overall, the main latent-state analysis used a prespecified six-state solution for exploratory characterization of recurrent ROI-level HbO activity patterns; each state exhibited distinct spatial activation patterns and temporal characteristics (Figure 3A), and group differences were observed between the healthy and stroke groups regarding the occupancy characteristics and dwell times of specific states (Figures 3C,D). In terms of spatial patterns, the various latent states displayed mutually distinguishable activation configurations across the 17 ROIs (Figure 3A). Specifically, State 1 was characterized by a broadly elevated positive activation pattern; States 2 and 5 were predominantly defined by overall negative activation patterns; State 4 exhibited a moderate level of widespread positive activation; while States 3 and 6 fell somewhere between these extremes. Representative state sequences from one participant in each group are shown in Figure 3B to illustrate the temporal ordering of latent states during the task. Because these sequences represent individual examples rather than group-level summaries, no inference regarding transition frequency or between-group differences was made from this panel. Group-level state characteristics were evaluated using fractional occupancy, mean dwell time, and transition probability matrices. Regarding fractional occupancy (FO) (Figure 3C), the distribution across the various states differed between the two groups. The healthy group exhibited higher occupancy rates for States 1 and 5, whereas the stroke group showed higher rates for States 3 and 6; notably, the inter-group differences for States 1, 3, 5, and 6 reached statistical significance, while no significant differences were observed for States 2 and 4. Based on the group mean values, the healthy group tended to occupy State 1 (FO mean = 0.0922) and State 5 (FO mean = 0.1315), whereas the stroke group tended to occupy State 3 (FO mean = 0.1887) and State 6 (FO mean = 0.2055). Regarding the mean dwell time (MDT) (Figure 3D), the stroke group exhibited longer durations in certain states. Specifically, the MDT for State 2 and State 3 was significantly higher in the stroke group than in the healthy group, while no significant inter-group differences were observed for the remaining states. Consistent with the group mean results, the stroke group demonstrated longer state maintenance durations in State 2 (7.5170 s vs. 6.0600 s) and State 3 (8.2612 s vs. 4.7760 s); furthermore, State 6 also exhibited a relatively long mean dwell time in the stroke group (11.1024 s), although the inter-group difference did not reach statistical significance. The transition matrices were dominated by diagonal elements in both groups (Figure 3E), indicating a relatively high probability of remaining in the same state between adjacent time points. A further inter-group difference matrix demonstrated that the disparities between the two groups were primarily concentrated in a few diagonal elements and specific off-diagonal transitions (Figure 3F). Notably, the healthy group exhibited relatively higher probabilities of self-maintenance in certain states, whereas the stroke group demonstrated a stronger tendency toward sustained occupancy in other states. The extended AIC/BIC model-comparison results and state-quality indices are provided in Supplementary Datasheet 7.

**Figure 3:**
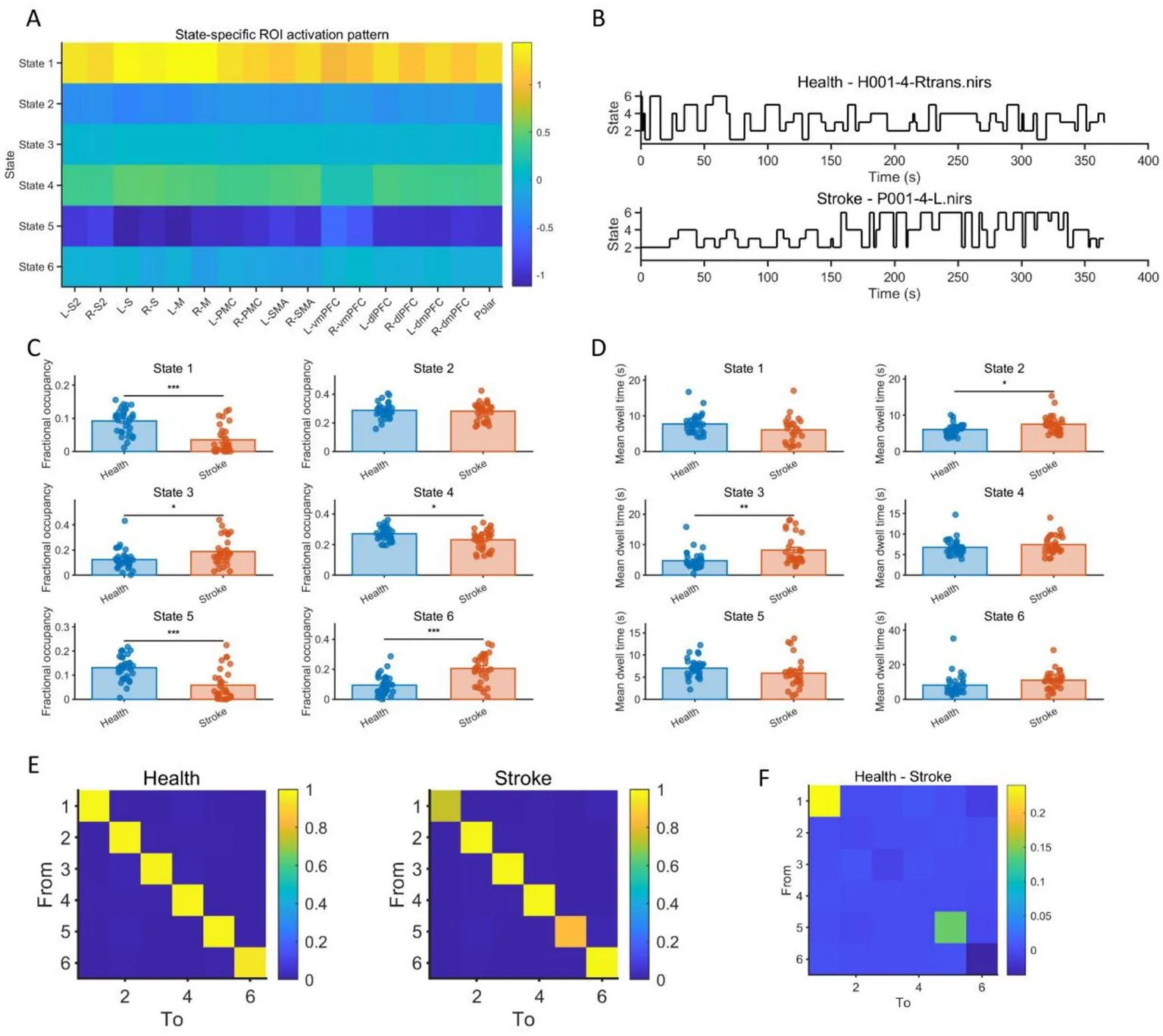
Comparison of latent state patterns and dynamic characteristics between the healthy group and the stroke group. **(A)** Spatial patterns of the six latent states derived from z-scored ROI-level HbO time series. Color values represent standardized HbO activation. **(B)** State sequences from one randomly selected participant in each group. These sequences are presented for descriptive visualization only and were not used to infer transition frequency or between-group differences. **(C)** Group comparison of the fractional occupancy (FO) for each latent state; each scatter point represents a single subject, bars indicate group means, and error bars represent standard errors; asterisks above the bars denote the significance of inter-group differences. **(D)** Group comparison of the mean dwell time (MDT) for each latent state; the graphical elements carry the same meaning as in (C). **(E)** Average state transition matrices for the healthy and stroke groups; the y-axis represents the current state (“From”), the x-axis represents the subsequent state (“To”), and colors indicate the average transition probabilities. **(F)** Difference map of the state transition matrices between the two groups (Health − Stroke), illustrating the spatial distribution of differences in state transition probabilities between the groups. ROI, region of interest; FO, fractional occupancy; MDT, mean dwell time.

### 3.4 Stroke patients show phase-specific reorganization of directed cortical information flow

The average directed connectivity heatmaps (Figure 4A) reveal that, in all three phases, both groups demonstrated dense connectivity among sensorimotor-related regions, as well as extensive non-zero directed connectivity among prefrontal-related regions and between these regions and the sensorimotor areas. In the healthy group, continuously distributed high-strength connectivity was observed across all three phases among bilateral S2, S, M, PMC, and SMA regions; similarly, the stroke group exhibited high average directed connectivity among sensorimotor-related nodes across the three phases, with certain prefrontal-related regions also participating in forming this continuous connectivity distribution. The inter-group difference network maps (Figure 4B) indicate that, during the pre-stimulation phase, differential connectivity was primarily distributed among bilateral sensory regions, PMC, SMA, and prefrontal-related regions; during the recovery phase, differential connectivity involved nodes such as the left sensory regions, SMA, PMC, vmPFC, dmPFC, and Polar; and during the stimulation phase, differential connectivity was predominantly distributed among the left S2, S, M, PMC, and SMA, as well as the right S2, vmPFC, dlPFC, and dmPFC regions. In all three phases, differential directed connectivity was observed both across hemispheres and between anterior and posterior regions. The dominant pathway migration trajectories (Figure 4C) demonstrate that the primary combinations of directed connectivity shifted across the different phases for both groups. For the healthy group, the dominant pathways during the pre-stimulation phase primarily included dlPFC→SMA, S → S2, PMC → SMA, dmPFC→SMA, SMA → vPMC, and S2 → SMA; during the stimulation phase, they primarily included S → PMC, M → PMC, dmPFC→PMC, PMC → PMC, and SMA → PMC; and during the recovery phase, they primarily included Polar→PMC, dlPFC→S2, vmPFC→SMA, S → S2, PMC → S2, and PMC → PMC. In the stroke group, the dominant pathways during the pre-stimulation phase primarily included vmPFC→S, M → S2, dlPFC→PMC, S → S2, PMC → S2, S2 → PMC, and SMA → S; during the stimulation phase, they mainly included SMA → S, PMC → S2, PMC → S, S2 → PMC, M → S, and dlPFC→S; and during the recovery phase, they primarily included SMA → S, dlPFC→S, Polar→PMC, Polar→S2, PMC → S, S → PMC, PMC → dmPFC, and dmPFC→PMC.

**Figure 4:**
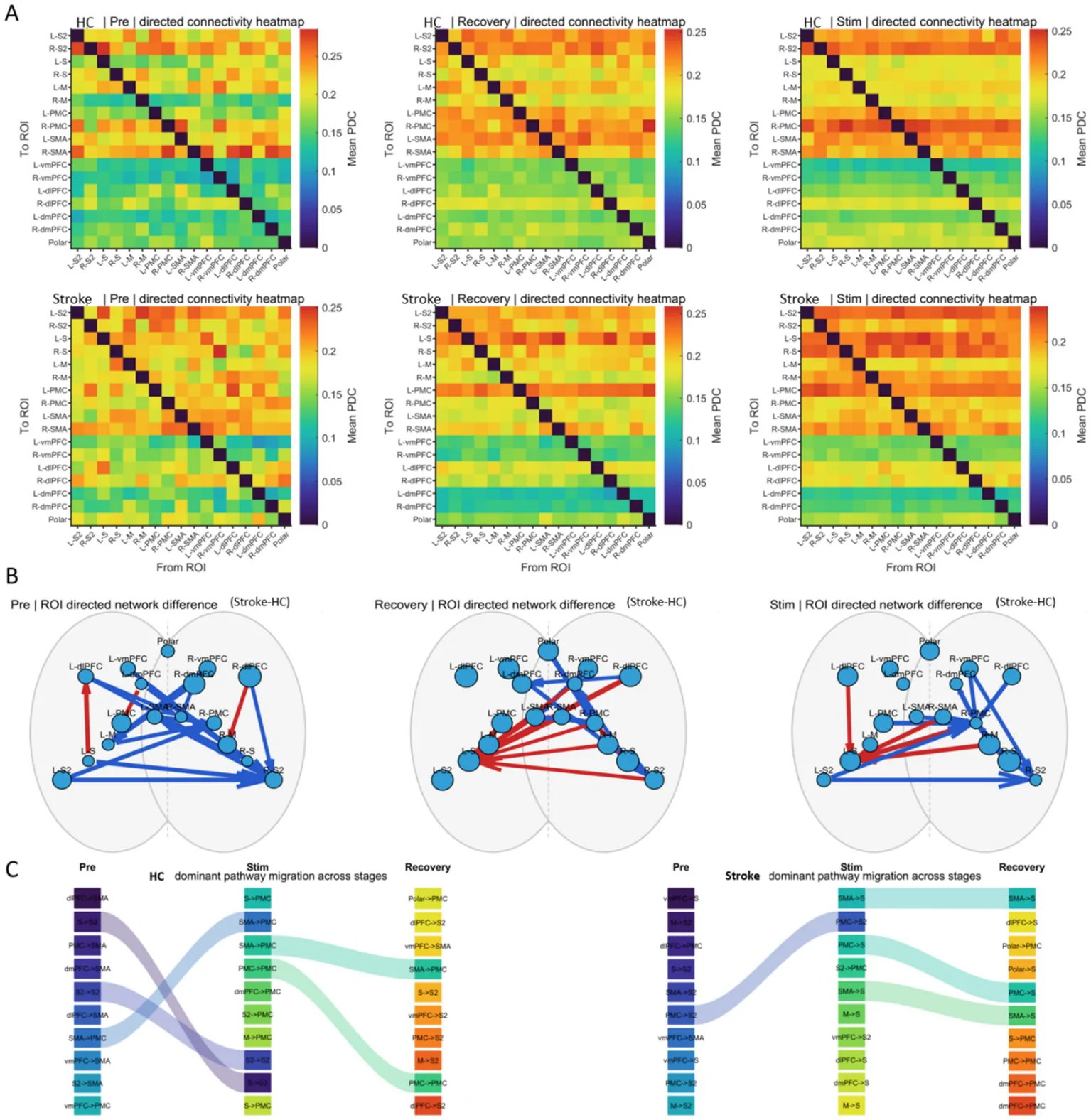
Comparison of ROI-level directed connectivity patterns and phase-specific transition characteristics between the healthy group and the stroke group. **(A)** Heatmaps of mean directed connectivity for the healthy and stroke groups during the pre-stimulation (Pre), recovery (Recovery), and stimulation (Stim) phases. The horizontal axis represents the source ROI (“From ROI”), the vertical axis represents the target ROI (“To ROI”), and the color intensity indicates the strength of the mean Partial Directed Coherence (PDC). **(B)** Maps illustrating inter-group differences in ROI-level directed networks across the three phases (Stroke − HC); arrow directions indicate the direction of information flow, with red arrows denoting connections that are stronger in the stroke group compared to the healthy group, and blue arrows denoting connections that are weaker in the stroke group. **(C)** Trajectories depicting the migration of dominant pathways across different phases for both groups, illustrating the changes in the primary directed connectivity configurations during the pre-stimulation, stimulation, and recovery phases. ROI, Region of Interest; PDC, Partial Directed Coherence; PMC, Premotor Cortex; SMA, Supplementary Motor Area; vmPFC, Ventromedial Prefrontal Cortex; dlPFC, Dorsolateral Prefrontal Cortex; dmPFC, Dorsomedial Prefrontal Cortex.

### 3.5 Stroke patients show more regular recovery-phase HbO dynamics

Figure 5 summarizes the recovery-phase complexity results. The multiscale entropy curves increased progressively from scale 1 to scale 5 in both groups (Figure 5A). Across all scales, the healthy group showed descriptively higher MSE values than the stroke group, although the group difference in global MeanMSE did not remain significant after FDR correction. The standardized difference heatmap showed a heterogeneous spatial distribution of group differences across the five complexity metrics and 17 ROIs (Figure 5B). Positive values indicate higher metric values in the stroke group, whereas negative values indicate higher values in the healthy group. The direction and magnitude of these differences varied across sensory, motor, premotor, supplementary motor, and prefrontal regions. However, none of the ROI-level comparisons survived FDR correction. At the global level (Figure 5C), SampEn did not differ significantly between groups (Health: 0.101 ± 0.031; Stroke: 0.088 ± 0.026; raw *p* = 0.070, FDR-adjusted q = 0.070). MeanMSE was lower in the stroke group than in the healthy group before correction, but the difference did not remain significant after FDR correction (Health: 0.267 ± 0.059; Stroke: 0.236 ± 0.062; raw *p* = 0.044, FDR-adjusted q = 0.055). In contrast, the stroke group showed significantly higher RR (0.081 ± 0.015 vs. 0.075 ± 0.016; raw *p* = 0.023, FDR-adjusted q = 0.049), DET (0.962 ± 0.018 vs. 0.949 ± 0.026; raw *p* = 0.022, FDR-adjusted q = 0.049), and Lmean (10.321 ± 4.921 vs. 8.382 ± 2.537; raw *p* = 0.030, FDR-adjusted q = 0.049) than the healthy group.

**Figure 5:**
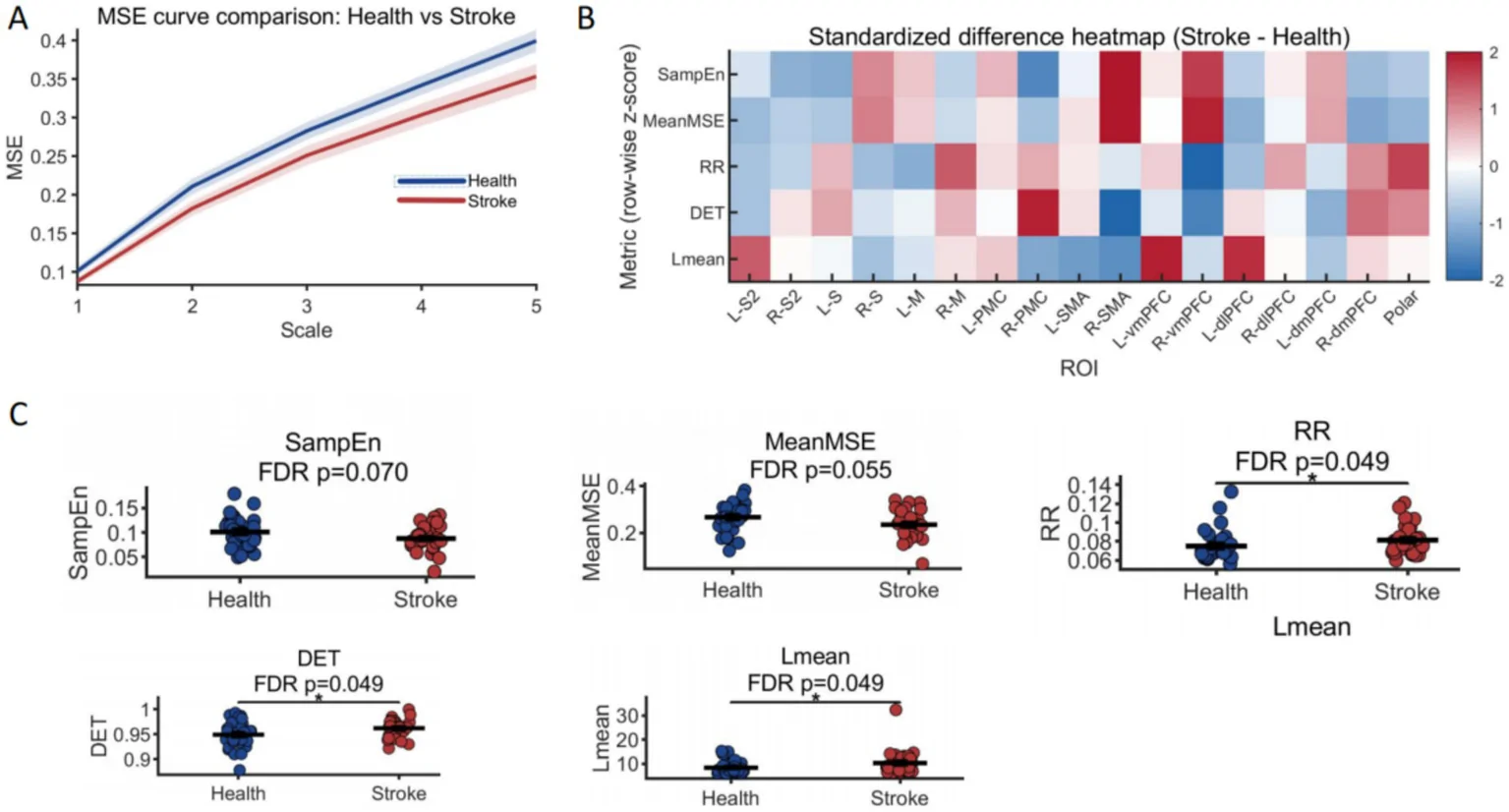
Recovery-phase complexity characteristics in the healthy and stroke groups. **(A)** Group-averaged multiscale entropy curves across scales 1–5; shaded areas indicate the standard error of the mean. **(B)** Heatmap of row-wise standardized between-group differences (Stroke − Health) in sample entropy (SampEn), mean multiscale entropy (MeanMSE), recurrence rate (RR), determinism (DET), and mean diagonal line length (Lmean) across 17 regions of interest. Red and blue indicate relatively higher values in the stroke and healthy groups, respectively. **(C)** Global between-group comparisons of SampEn, MeanMSE, RR, DET, and Lmean. Each point represents one participant, and horizontal markers and error bars indicate the group mean and standard error of the mean. FDR-adjusted *q* values are shown above each comparison.

## 4 Discussion

This study used fNIRS to record cortical HbO dynamics in patients with stroke and healthy controls during a passive thumb movement task and characterized abnormalities in cortical activity induced by passive proprioceptive-related stimulation after stroke from four perspectives: temporal features, latent states, directed connectivity, and recovery-phase complexity. The main findings showed that patients with stroke exhibited attenuated task-evoked HbO responses in sensorimotor-related regions and slower post-stimulus hemodynamic recovery. Full-time latent-state analysis indicated abnormal state occupancy and dwell characteristics in patients with stroke. Directed connectivity analysis showed that interregional information-flow patterns were reorganized across different task phases. Recovery-phase complexity analysis further suggested that post-stimulus HbO dynamics were more regular and repetitive in patients with stroke.

Abnormalities in HbO temporal features were mainly characterized by reduced response amplitudes in sensorimotor-related regions and flatter post-stimulus recovery slopes. Passive thumb movement predominantly induces afferent input related to joint position sense and kinesthesia; therefore, these findings may be interpreted as insufficient sensorimotor cortical responses and delayed post-stimulus return under passive proprioceptive-related stimulation in patients with stroke (18). Previous studies have shown that proprioception plays an important role in upper-limb motor control, online correction, and functional recovery (1, 19). Impairments in position sense and tactile discrimination are common after stroke and are associated with poorer motor performance (3, 4). In a longitudinal fMRI study, Ward et al. found that cerebral responses to proprioceptive input changed over the course of recovery after stroke (5). Ingemanson et al. also suggested that brain activity related to passive finger position sense was associated with proprioceptive deficits after stroke (6, 13, 20, 21). Thus, the abnormalities in HbO amplitude and recovery slope observed in the present study are broadly consistent with the established framework linking proprioceptive input, sensorimotor cortical responses, and upper-limb functional status. It should be emphasized that recovery slope in this study refers to the rate at which the post-stimulus HbO signal declines from its peak toward baseline, rather than the rate of clinical recovery after stroke. A flatter slope indicates slower decay of the task-evoked hemodynamic response and may reflect reduced regulatory efficiency of the sensorimotor cortex after stroke or prolonged post-stimulus responses. Recent work by Wu et al. on intrinsic neural timescales and criticality suggests that post-stroke brain dynamics may be characterized by prolonged timescales and slower activity decay, providing a conceptual clue for understanding abnormal dynamic recovery after stroke. However, the present study measured task-evoked HbO hemodynamic decay rather than intrinsic neural timescales directly; the two concepts should therefore be regarded as related but not equivalent (22). Latent-state analysis further showed abnormalities in state occupancy and dwell time throughout the full task period in patients with stroke. This finding suggests that cortical activity after stroke may be more likely to remain in certain recurrent hemodynamic activity patterns, rather than exhibiting the same flexible state organization observed in healthy controls during task performance. Using sliding-window dynamic functional connectivity, Bonkhoff et al. found that acute stroke altered the brain’s preference for different dynamic connectivity states and that dynamic connectivity features could predict acute motor impairment and subsequent recovery (9, 23, 24). A review by Wu et al. on static and dynamic functional connectivity after stroke also noted that dynamic brain network analysis can complement static connectivity measures by revealing time-varying network reorganization associated with post-stroke motor dysfunction and recovery (10, 21). However, the latent states identified in the present study were not sliding-window functional connectivity states in the conventional sense, but cortical hemodynamic activity states derived by clustering ROI-level HbO activation patterns. Therefore, these findings are more appropriately interpreted as abnormalities in the organization of cortical activity states throughout the passive thumb movement task, rather than as abnormalities in dynamic functional connectivity states in the strict sense.

Directed connectivity analysis showed that healthy controls and patients with stroke exhibited different ROI-to-ROI information-flow patterns during the pre-stimulation, stimulation, and recovery phases. PDC provides a methodological framework for exploring directional coupling among multiregional time series, and previous fNIRS studies have applied time-frequency effective connectivity to describe dynamic changes in human brain networks during task performance (25–27). The phase-dependent reorganization of directed information flow observed in this study suggests that passive proprioceptive-related stimulation may alter not only local cortical responses but also the coordinated relationships among sensorimotor, premotor, supplementary motor, and prefrontal regions. This interpretation is consistent with the network framework of stroke recovery proposed by Cassidy and Cramer, according to which post-stroke dysfunction and recovery should not be understood solely as local lesion effects but should instead be interpreted in the context of distributed functional connectivity and network reorganization (7). Nevertheless, the PDC findings should be interpreted cautiously. The present study used a 20-s sliding window and band-averaged PDC within the 0.01–0.08 Hz range, primarily to describe phase-dependent directional coupling patterns rather than to estimate neural causal effects at a specific frequency with precision. Accordingly, these findings are interpreted as exploratory directed coupling patterns rather than definitive causal pathways. Recovery-phase complexity analysis showed increased RR, DET, and Lmean in patients with stroke, suggesting that post-stimulus HbO signals may exhibit greater repetitiveness, determinism, and regularity. Complex physiological systems generally need to maintain a balance between stability and flexibility, and entropy and recurrence quantification analyses can be used to characterize the nonlinear organization of time series (15–17). In the context of the present study, increased recovery-phase complexity does not necessarily indicate better function. Instead, it may suggest that post-stimulus hemodynamic activity has greater difficulty returning rapidly to a flexible baseline fluctuation pattern and therefore remains in a more regular and sustained dynamic mode (28). This interpretation is partly consistent with the flatter recovery slope and abnormal latent-state dwell characteristics described above, collectively pointing to an abnormal post-task cortical dynamic recovery process after stroke. However, because the recovery-phase time window was relatively short, these complexity findings should be regarded as exploratory and require validation using longer task recordings and independent samples.

This study has several limitations. The sample size was relatively limited, and some findings were close to the threshold for statistical significance. Non-significant results should not be interpreted simply as evidence of no abnormality. In particular, the absence of significant differences in onset latency and time-to-peak may have been related to insufficient statistical power. Manual passive thumb movement was used as a proprioceptive-related stimulus. Although non-target input was reduced by asking participants to keep their eyes closed, minimizing active movement, and standardizing therapist operation, skin contact, stretching, and mild tactile input could not be completely excluded. Therefore, the present findings should be interpreted as cortical dynamic abnormalities induced by passive proprioceptive-related stimulation rather than as direct evidence of a pure proprioceptive processing disorder. Patients with stroke also differed in lesion side. Hemisphere standardization or affected-side alignment facilitates group-level analysis but may obscure intrinsic left–right hemispheric differences and heterogeneity associated with different lesion locations (29). the recovery-phase complexity analysis was based on a relatively short time window, which may have affected the stability of the entropy and RQA measures (24, 30). This study integrated multiple fNIRS dynamic analysis methods to provide exploratory multidimensional evidence, but the clinical significance of these measures still requires further validation in larger samples, with tactile-matched control conditions, online behavioral assessment of position sense, longitudinal follow-up, and multimodal EEG/fMRI data (31, 32).

In conclusion, this study suggests that patients with stroke exhibit multilevel abnormalities in cortical dynamics during passive proprioceptive-related stimulation, including attenuated HbO responses in sensorimotor regions, delayed post-stimulus hemodynamic return, abnormal organization of cortical activity states, phase-dependent reorganization of directed information flow, and increased regularity of recovery-phase dynamics. The advantages of fNIRS include its portability, good tolerability, and suitability for continuous monitoring of brain function during task performance in clinical stroke populations. Future studies should further combine standardized robot-controlled passive movement, tactile control tasks, and longitudinal clinical outcomes to determine whether these fNIRS-derived dynamic measures can serve as interpretable biomarkers of sensorimotor circuit dysfunction and rehabilitation response after stroke.

## Data Availability

The original contributions presented in the study are included in the article/Supplementary material, further inquiries can be directed to the corresponding author.
