# Supplementary material for: Attenuated sensorimotor hemodynamic responses and altered cortical dynamics during passive proprioceptive-related stimulation after stroke: a multidimensional fNIRS study
Source: Front Neurol. 2026 Sep 16;17:1848276. doi: 10.3389/fneur.2026.1848276 (PMC13624074; doi:10.3389/fneur.2026.1848276)
Supplement: Supplementary file 1 [file Data_Sheet_3.DOCX]

89 C	ha	nnels and 52 Optodes

"# ,"	A	"natomical label, Percentage of Overlap"

CH01	:	"10 - Frontopolar area, 0.69314"

CH01	:	"11 - Orbitofrontal area, 0.28881"

CH01	:	"46 - Dorsolateral prefrontal cortex, 0.01444"

CH01	:	"47 - Inferior prefrontal gyrus, 0.0036101"

CH02	:	"10 - Frontopolar area, 0.80645"

CH02	:	"11 - Orbitofrontal area, 0.19355"

CH03	:	"10 - Frontopolar area, 0.83596"

CH03	:	"11 - Orbitofrontal area, 0.16404"

CH04	:	"10 - Frontopolar area, 0.6121"

CH04	:	"11 - Orbitofrontal area, 0.36655"

CH04	:	"46 - Dorsolateral prefrontal cortex, 0.00355"	87

CH04	:	"47 - Inferior prefrontal gyrus, 0.017794"

CH05	:	"45 - pars triangularis Broca's area, 0.09454"	5

CH05	:	"46 - Dorsolateral prefrontal cortex, 0.83273"

CH05	:	"47 - Inferior prefrontal gyrus, 0.072727"

CH06	:	"10 - Frontopolar area, 0.16342"

CH06	:	"45 - pars triangularis Broca's area, 0.01556"	4

CH06	:	"46 - Dorsolateral prefrontal cortex, 0.82101"

CH07	:	"10 - Frontopolar area, 0.99643"

CH07	:	"46 - Dorsolateral prefrontal cortex, 0.00357"	14

CH08	:	"10 - Frontopolar area, 1"

CH09	:	"10 - Frontopolar area, 1"

CH10	:	"10 - Frontopolar area, 0.21094"

CH10	:	"46 - Dorsolateral prefrontal cortex, 0.78906"

CH11	:	"45 - pars triangularis Broca's area, 0.03237"	4

CH11	:	"46 - Dorsolateral prefrontal cortex, 0.8741"

CH11	:	"47 - Inferior prefrontal gyrus, 0.093525"

CH12	:	"10 - Frontopolar area, 0.03719"

CH12	:	"46 - Dorsolateral prefrontal cortex, 0.96281"

CH13	:	"9 - Dorsolateral prefrontal cortex, 0.16846"

CH13	:	"10 - Frontopolar area, 0.82437"

CH13	:	"46 - Dorsolateral prefrontal cortex, 0.00716"	85

CH14	:	"9 - Dorsolateral prefrontal cortex, 0.076642"

CH14	:	"10 - Frontopolar area, 0.92336"

CH15	:	"10 - Frontopolar area, 0.1308"

CH15	:	"46 - Dorsolateral prefrontal cortex, 0.8692"

CH16	:	"45 - pars triangularis Broca's area, 0.95098"

CH16	:	"46 - Dorsolateral prefrontal cortex, 0.04902"

CH17	:	"45 - pars triangularis Broca's area, 0.87636"

CH17	:	"46 - Dorsolateral prefrontal cortex, 0.12364"

CH18	:	"9 - Dorsolateral prefrontal cortex, 0.03913"

CH18	:	"45 - pars triangularis Broca's area, 0.56087"

CH18	:	"46 - Dorsolateral prefrontal cortex, 0.4"

CH19	:	"9 - Dorsolateral prefrontal cortex, 0.57143"

CH19	:	"10 - Frontopolar area, 0.04329"

CH19	:	"46 - Dorsolateral prefrontal cortex, 0.38528"

CH20	:	"9 - Dorsolateral prefrontal cortex, 0.72619"

CH20	:	"10 - Frontopolar area, 0.27381"

CH21	:	"9 - Dorsolateral prefrontal cortex, 0.67094"

CH21	:	"10 - Frontopolar area, 0.07265"

CH21	:	"46 - Dorsolateral prefrontal cortex, 0.25641"

CH22	:	"9 - Dorsolateral prefrontal cortex, 0.053097"

CH22	:	"45 - pars triangularis Broca's area, 0.5354"

CH22	:	"46 - Dorsolateral prefrontal cortex, 0.4115"

CH23	:	"45 - pars triangularis Broca's area, 0.80515"

CH23	:	"46 - Dorsolateral prefrontal cortex, 0.19485"

CH24	:	"45 - pars triangularis Broca's area, 0.91148"

CH24	:	"46 - Dorsolateral prefrontal cortex, 0.08852"	5

CH25	:	"38 - Temporopolar area, 0.7953"

CH25	:	"45 - pars triangularis Broca's area, 0.14094"

CH25	:	"47 - Inferior prefrontal gyrus, 0.063758"

CH26	:	"9 - Dorsolateral prefrontal cortex, 0.86996"

CH26	:	"46 - Dorsolateral prefrontal cortex, 0.13004"

CH27	:	"9 - Dorsolateral prefrontal cortex, 1"

CH28	:	"8 - Includes Frontal eye fields, 0.0041322"

CH28	:	"9 - Dorsolateral prefrontal cortex, 0.99587"

CH29	:	"9 - Dorsolateral prefrontal cortex, 0.96861"

CH29	:	"46 - Dorsolateral prefrontal cortex, 0.03139"

CH30	:	"38 - Temporopolar area, 0.73826"

CH30	:	"45 - pars triangularis Broca's area, 0.1745"

CH30	:	"47 - Inferior prefrontal gyrus, 0.087248"

CH31	:	"6 - Pre-Motor and Supplementary Motor Cortex,"	0.019169

CH31	:	"44 - pars opercularis, part of Broca's area,"	0.3738

CH31	:	"45 - pars triangularis Broca's area, 0.57188"

CH31	:	"48 - Retrosubicular area, 0.035144"

CH32	:	"6 - Pre-Motor and Supplementary Motor Cortex,"	0.042017

CH32	:	"9 - Dorsolateral prefrontal cortex, 0.90336"

CH32	:	"44 - pars opercularis, part of Broca's area,"	0.054622

CH33	:	"8 - Includes Frontal eye fields, 0.57025"

CH33	:	"9 - Dorsolateral prefrontal cortex, 0.42975"

CH34	:	"8 - Includes Frontal eye fields, 0.8797"

CH34	:	"9 - Dorsolateral prefrontal cortex, 0.1203"

CH35	:	"8 - Includes Frontal eye fields, 0.65339"

CH35	:	"9 - Dorsolateral prefrontal cortex, 0.34661"

CH36	:	"8 - Includes Frontal eye fields, 0.0084388"

CH36	:	"9 - Dorsolateral prefrontal cortex, 0.95359"

CH36	:	"44 - pars opercularis, part of Broca's area,"	0.037975

CH37	:	"44 - pars opercularis, part of Broca's area,"	0.28155

CH37	:	"45 - pars triangularis Broca's area, 0.71197"

CH37	:	"48 - Retrosubicular area, 0.0064725"

CH38	:	"6 - Pre-Motor and Supplementary Motor Cortex,"	0.071429

CH38	:	"21 - Middle Temporal gyrus, 0.10204"

CH38	:	"38 - Temporopolar area, 0.27551"

CH38	:	"48 - Retrosubicular area, 0.55102"

CH39	:	"6 - Pre-Motor and Supplementary Motor Cortex,"	0.57785

CH39	:	"43 - Subcentral area, 0.11073"

CH39	:	"44 - pars opercularis, part of Broca's area,"	0.31142

CH40	:	"6 - Pre-Motor and Supplementary Motor Cortex,"	0.65517

CH40	:	"9 - Dorsolateral prefrontal cortex, 0.29502"

CH40	:	"44 - pars opercularis, part of Broca's area,"	0.049808

CH41	:	"6 - Pre-Motor and Supplementary Motor Cortex,"	0.14844

CH41	:	"8 - Includes Frontal eye fields, 0.53516"

CH41	:	"9 - Dorsolateral prefrontal cortex, 0.31641"

CH42	:	"6 - Pre-Motor and Supplementary Motor Cortex,"	0.11388

CH42	:	"8 - Includes Frontal eye fields, 0.88612"

CH43	:	"6 - Pre-Motor and Supplementary Motor Cortex,"	0.10105

CH43	:	"8 - Includes Frontal eye fields, 0.89895"

CH44	:	"6 - Pre-Motor and Supplementary Motor Cortex,"	0.045627

CH44	:	"8 - Includes Frontal eye fields, 0.61217"

CH44	:	"9 - Dorsolateral prefrontal cortex, 0.34221"

CH45	:	"6 - Pre-Motor and Supplementary Motor Cortex,"	0.66932

CH45	:	"9 - Dorsolateral prefrontal cortex, 0.31474"

CH45	:	"44 - pars opercularis, part of Broca's area,"	0.015936

CH46	:	"4 - Primary Motor Cortex, 0.010309"

CH46	:	"6 - Pre-Motor and Supplementary Motor Cortex,"	0.63574

CH46	:	"43 - Subcentral area, 0.13746"

CH46	:	"44 - pars opercularis, part of Broca's area,"	0.21649

CH47	:	"6 - Pre-Motor and Supplementary Motor Cortex,"	0.067568

CH47	:	"21 - Middle Temporal gyrus, 0.12162"

CH47	:	"38 - Temporopolar area, 0.30068"

CH47	:	"48 - Retrosubicular area, 0.51014"

CH48	:	"21 - Middle Temporal gyrus, 1"

CH49	:	"6 - Pre-Motor and Supplementary Motor Cortex,"	0.097484

CH49	:	"22 - Superior Temporal Gyrus, 0.18868"

CH49	:	"43 - Subcentral area, 0.54403"

CH49	:	"48 - Retrosubicular area, 0.16981"

CH50	:	"1 - Primary Somatosensory Cortex, 0.066901"

CH50	:	"3 - Primary Somatosensory Cortex, 0.15141"

CH50	:	"4 - Primary Motor Cortex, 0.29577"

CH50	:	"6 - Pre-Motor and Supplementary Motor Cortex,"	0.25704

CH50	:	"43 - Subcentral area, 0.22887"

CH51	:	"3 - Primary Somatosensory Cortex, 0.004"

CH51	:	"4 - Primary Motor Cortex, 0.332"

CH51	:	"6 - Pre-Motor and Supplementary Motor Cortex,"	0.664

CH52	:	"6 - Pre-Motor and Supplementary Motor Cortex,"	0.6782

CH52	:	"8 - Includes Frontal eye fields, 0.3218"

CH53	:	"6 - Pre-Motor and Supplementary Motor Cortex,"	0.9375

CH53	:	"8 - Includes Frontal eye fields, 0.0625"

CH54	:	"6 - Pre-Motor and Supplementary Motor Cortex,"	0.6263

CH54	:	"8 - Includes Frontal eye fields, 0.3737"

CH55	:	"3 - Primary Somatosensory Cortex, 0.0040323"

CH55	:	"4 - Primary Motor Cortex, 0.41129"

CH55	:	"6 - Pre-Motor and Supplementary Motor Cortex,"	0.58468

CH56	:	"1 - Primary Somatosensory Cortex, 0.039568"

CH56	:	"3 - Primary Somatosensory Cortex, 0.16906"

CH56	:	"4 - Primary Motor Cortex, 0.27698"

CH56	:	"6 - Pre-Motor and Supplementary Motor Cortex,"	0.33094

CH56	:	"43 - Subcentral area, 0.18345"

CH57	:	"6 - Pre-Motor and Supplementary Motor Cortex,"	0.069401

CH57	:	"22 - Superior Temporal Gyrus, 0.21767"

CH57	:	"43 - Subcentral area, 0.5836"

CH57	:	"48 - Retrosubicular area, 0.12934"

CH58	:	"21 - Middle Temporal gyrus, 1"

CH59	:	"21 - Middle Temporal gyrus, 0.60703"

CH59	:	"22 - Superior Temporal Gyrus, 0.39297"

CH60	:	"1 - Primary Somatosensory Cortex, 0.19231"

CH60	:	"2 - Primary Somatosensory Cortex, 0.50962"

CH60	:	"43 - Subcentral area, 0.24359"

CH60	:	"48 - Retrosubicular area, 0.054487"

CH61	:	"1 - Primary Somatosensory Cortex, 0.2621"

CH61	:	"3 - Primary Somatosensory Cortex, 0.55645"

CH61	:	"4 - Primary Motor Cortex, 0.18145"

CH62	:	"4 - Primary Motor Cortex, 0.35448"

CH62	:	"6 - Pre-Motor and Supplementary Motor Cortex,"	0.64552

CH63	:	"6 - Pre-Motor and Supplementary Motor Cortex,"	1

CH64	:	"6 - Pre-Motor and Supplementary Motor Cortex,"	1

CH65	:	"4 - Primary Motor Cortex, 0.2952"

CH65	:	"6 - Pre-Motor and Supplementary Motor Cortex,"	0.7048

CH66	:	"1 - Primary Somatosensory Cortex, 0.37154"

CH66	:	"3 - Primary Somatosensory Cortex, 0.44664"

CH66	:	"4 - Primary Motor Cortex, 0.18182"

CH67	:	"1 - Primary Somatosensory Cortex, 0.23197"

CH67	:	"2 - Primary Somatosensory Cortex, 0.5674"

CH67	:	"43 - Subcentral area, 0.20063"

CH68	:	"21 - Middle Temporal gyrus, 0.52201"

CH68	:	"22 - Superior Temporal Gyrus, 0.47799"

CH69	:	"20 - Inferior Temporal gyrus, 0.28125"

CH69	:	"21 - Middle Temporal gyrus, 0.71875"

CH70	:	"2 - Primary Somatosensory Cortex, 0.10703"

CH70	:	"22 - Superior Temporal Gyrus, 0.69419"

CH70	:	"42 - Primary and Auditory Association Cortex,"	0.061162

CH70	:	"48 - Retrosubicular area, 0.13761"

CH71	:	"1 - Primary Somatosensory Cortex, 0.041812"

CH71	:	"2 - Primary Somatosensory Cortex, 0.26829"

CH71	:	"3 - Primary Somatosensory Cortex, 0.013937"

CH71	:	40 - Supramarginal gyrus part of Wernicke's a	"rea, 0.67596"

CH72	:	"1 - Primary Somatosensory Cortex, 0.25292"

CH72	:	"2 - Primary Somatosensory Cortex, 0.25681"

CH72	:	"3 - Primary Somatosensory Cortex, 0.36965"

CH72	:	"4 - Primary Motor Cortex, 0.11284"

CH72	:	40 - Supramarginal gyrus part of Wernicke's a	"rea, 0.0077821"

CH73	:	"4 - Primary Motor Cortex, 0.26623"

CH73	:	"6 - Pre-Motor and Supplementary Motor Cortex,"	0.73377

CH74	:	"4 - Primary Motor Cortex, 0.29474"

CH74	:	"6 - Pre-Motor and Supplementary Motor Cortex,"	0.70526

CH75	:	"4 - Primary Motor Cortex, 0.27922"

CH75	:	"6 - Pre-Motor and Supplementary Motor Cortex,"	0.72078

CH76	:	"1 - Primary Somatosensory Cortex, 0.2"

CH76	:	"2 - Primary Somatosensory Cortex, 0.23846"

CH76	:	"3 - Primary Somatosensory Cortex, 0.42692"

CH76	:	"4 - Primary Motor Cortex, 0.13462"

CH77	:	"1 - Primary Somatosensory Cortex, 0.21053"

CH77	:	"2 - Primary Somatosensory Cortex, 0.094737"

CH77	:	40 - Supramarginal gyrus part of Wernicke's a	"rea, 0.69474"

CH78	:	"2 - Primary Somatosensory Cortex, 0.090343"

CH78	:	"22 - Superior Temporal Gyrus, 0.73209"

CH78	:	40 - Supramarginal gyrus part of Wernicke's a	"rea, 0.084112"

CH78	:	"48 - Retrosubicular area, 0.093458"

CH79	:	"20 - Inferior Temporal gyrus, 0.28435"

CH79	:	"21 - Middle Temporal gyrus, 0.71565"

CH80	:	"20 - Inferior Temporal gyrus, 0.08125"

CH80	:	"21 - Middle Temporal gyrus, 0.47187"

CH80	:	"22 - Superior Temporal Gyrus, 0.42188"

CH80	:	"37 - Fusiform gyrus, 0.025"

CH81	:	"22 - Superior Temporal Gyrus, 0.1619"

CH81	:	"39 - Angular gyrus, part of Wernicke's area,"	0.0031746

CH81	:	40 - Supramarginal gyrus part of Wernicke's a	"rea, 0.6254"

CH81	:	"48 - Retrosubicular area, 0.20952"

CH82	:	40 - Supramarginal gyrus part of Wernicke's a	"rea, 1"

CH83	:	"1 - Primary Somatosensory Cortex, 0.29123"

CH83	:	"2 - Primary Somatosensory Cortex, 0.15439"

CH83	:	"3 - Primary Somatosensory Cortex, 0.24211"

CH83	:	"4 - Primary Motor Cortex, 0.080702"

CH83	:	"7 - Somatosensory Association Cortex, 0.2140"	4

CH83	:	40 - Supramarginal gyrus part of Wernicke's a	"rea, 0.017544"

CH84	:	"1 - Primary Somatosensory Cortex, 0.059561"

CH84	:	"3 - Primary Somatosensory Cortex, 0.17555"

CH84	:	"4 - Primary Motor Cortex, 0.73981"

CH84	:	"5 - Somatosensory Association Cortex, 0.0250"	78

CH85	:	"1 - Primary Somatosensory Cortex, 0.040498"

CH85	:	"3 - Primary Somatosensory Cortex, 0.15576"

CH85	:	"4 - Primary Motor Cortex, 0.77882"

CH85	:	"5 - Somatosensory Association Cortex, 0.0249"	22

CH86	:	"1 - Primary Somatosensory Cortex, 0.28159"

CH86	:	"2 - Primary Somatosensory Cortex, 0.097473"

CH86	:	"3 - Primary Somatosensory Cortex, 0.26354"

CH86	:	"4 - Primary Motor Cortex, 0.068592"

CH86	:	"7 - Somatosensory Association Cortex, 0.2599"	3

CH86	:	40 - Supramarginal gyrus part of Wernicke's a	"rea, 0.028881"

CH87	:	"2 - Primary Somatosensory Cortex, 0.0076628"

CH87	:	40 - Supramarginal gyrus part of Wernicke's a	"rea, 0.99234"

CH88	:	"22 - Superior Temporal Gyrus, 0.14935"

CH88	:	"39 - Angular gyrus, part of Wernicke's area,"	0.0032468

CH88	:	40 - Supramarginal gyrus part of Wernicke's a	"rea, 0.72403"

CH88	:	"48 - Retrosubicular area, 0.12338"

CH89	:	"20 - Inferior Temporal gyrus, 0.079618"

CH89	:	"21 - Middle Temporal gyrus, 0.43631"

CH89	:	"22 - Superior Temporal Gyrus, 0.4586"

CH89	:	"37 - Fusiform gyrus, 0.025478"

S1 :	1	"0 - Frontopolar area, 0.304"

S1 :	4	"6 - Dorsolateral prefrontal cortex, 0.676"

S1 :	4	"7 - Inferior prefrontal gyrus, 0.02"

D1 :	1	"0 - Frontopolar area, 0.57667"

D1 :	1	"1 - Orbitofrontal area, 0.42333"

S2 :	1	"0 - Frontopolar area, 1"

D2 :	1	"0 - Frontopolar area, 0.557"

D2 :	1	"1 - Orbitofrontal area, 0.443"

S3 :	1	"0 - Frontopolar area, 0.38866"

S3 :	4	"6 - Dorsolateral prefrontal cortex, 0.60324"

S3 :	4	"7 - Inferior prefrontal gyrus, 0.0080972"

D4 :	4	"5 - pars triangularis Broca's area, 0.40562"

D4 :	4	"6 - Dorsolateral prefrontal cortex, 0.59438"

S4 :	9	"- Dorsolateral prefrontal cortex, 0.054902"

S4 :	1	"0 - Frontopolar area, 0.54118"

S4 :	4	"6 - Dorsolateral prefrontal cortex, 0.40392"

D5 :	9	"- Dorsolateral prefrontal cortex, 0.051852"

D5 :	1	"0 - Frontopolar area, 0.94815"

S5 :	9	"- Dorsolateral prefrontal cortex, 0.089844"

S5 :	1	"0 - Frontopolar area, 0.62891"

S5 :	4	"6 - Dorsolateral prefrontal cortex, 0.28125"

D6 :	4	"5 - pars triangularis Broca's area, 0.32636"

D6 :	4	"6 - Dorsolateral prefrontal cortex, 0.67364"

D3 :	3	"8 - Temporopolar area, 0.024138"

D3 :	4	"5 - pars triangularis Broca's area, 0.42414"

D3 :	4	"6 - Dorsolateral prefrontal cortex, 0.31379"

D3 :	4	"7 - Inferior prefrontal gyrus, 0.23793"

S6 :	4	"5 - pars triangularis Broca's area, 1"

S7 :	9	"- Dorsolateral prefrontal cortex, 0.49776"

S7 :	4	"4 - pars opercularis, part of Broca's area, 0"	0.18386

S7 :	4	"5 - pars triangularis Broca's area, 0.20179"

S7 :	4	"6 - Dorsolateral prefrontal cortex, 0.11659"

D8 :	9	"- Dorsolateral prefrontal cortex, 1"

S8 :	8	"- Includes Frontal eye fields, 0.16596"

S8 :	9	"- Dorsolateral prefrontal cortex, 0.83404"

D9 :	9	"- Dorsolateral prefrontal cortex, 1"

S9 :	9	"- Dorsolateral prefrontal cortex, 0.61161"

S9 :	4	"4 - pars opercularis, part of Broca's area, 0"	0.13393

S9 :	4	"5 - pars triangularis Broca's area, 0.13839"

S9 :	4	"6 - Dorsolateral prefrontal cortex, 0.11607"

S10	:	"45 - pars triangularis Broca's area, 1"

D7 :	3	"8 - Temporopolar area, 0.020067"

D7 :	4	"5 - pars triangularis Broca's area, 0.36789"

D7 :	4	"6 - Dorsolateral prefrontal cortex, 0.34783"

D7 :	4	"7 - Inferior prefrontal gyrus, 0.26421"

S11	:	"21 - Middle Temporal gyrus, 0.6958"

S11	:	"38 - Temporopolar area, 0.3042"

D10	:	"6 - Pre-Motor and Supplementary Motor Cortex,"	0.56818

D10	:	"43 - Subcentral area, 0.022727"

D10	:	"44 - pars opercularis, part of Broca's area,"	0.28247

D10	:	"48 - Retrosubicular area, 0.12662"

S12	:	"4 - Primary Motor Cortex, 0.04059"

S12	:	"6 - Pre-Motor and Supplementary Motor Cortex,"	0.66052

S12	:	"9 - Dorsolateral prefrontal cortex, 0.0073801"

S12	:	"44 - pars opercularis, part of Broca's area,"	0.29151

D11	:	"6 - Pre-Motor and Supplementary Motor Cortex,"	0.69136

D11	:	"8 - Includes Frontal eye fields, 0.057613"

D11	:	"9 - Dorsolateral prefrontal cortex, 0.25103"

S13	:	"6 - Pre-Motor and Supplementary Motor Cortex,"	0.030189

S13	:	"8 - Includes Frontal eye fields, 0.96981"

D12	:	"6 - Pre-Motor and Supplementary Motor Cortex,"	0.073529

D12	:	"8 - Includes Frontal eye fields, 0.92647"

S14	:	"6 - Pre-Motor and Supplementary Motor Cortex,"	0.0037175

S14	:	"8 - Includes Frontal eye fields, 0.99628"

D13	:	"6 - Pre-Motor and Supplementary Motor Cortex,"	0.5743

D13	:	"8 - Includes Frontal eye fields, 0.048193"

D13	:	"9 - Dorsolateral prefrontal cortex, 0.37751"

S15	:	"4 - Primary Motor Cortex, 0.075758"

S15	:	"6 - Pre-Motor and Supplementary Motor Cortex,"	0.69697

S15	:	"44 - pars opercularis, part of Broca's area,"	0.22727

D14	:	"6 - Pre-Motor and Supplementary Motor Cortex,"	0.60518

D14	:	"43 - Subcentral area, 0.038835"

D14	:	"44 - pars opercularis, part of Broca's area,"	0.26214

D14	:	"48 - Retrosubicular area, 0.093851"

S16	:	"21 - Middle Temporal gyrus, 0.67698"

S16	:	"38 - Temporopolar area, 0.32302"

D15	:	"20 - Inferior Temporal gyrus, 0.047923"

D15	:	"21 - Middle Temporal gyrus, 0.94249"

D15	:	"22 - Superior Temporal Gyrus, 0.0095847"

S17	:	"2 - Primary Somatosensory Cortex, 0.097561"

S17	:	"22 - Superior Temporal Gyrus, 0.57622"

S17	:	"43 - Subcentral area, 0.21951"

S17	:	"48 - Retrosubicular area, 0.10671"

D16	:	"1 - Primary Somatosensory Cortex, 0.3614"

D16	:	"2 - Primary Somatosensory Cortex, 0.24561"

D16	:	"3 - Primary Somatosensory Cortex, 0.34386"

D16	:	"4 - Primary Motor Cortex, 0.049123"

S18	:	"1 - Primary Somatosensory Cortex, 0.083969"

S18	:	"3 - Primary Somatosensory Cortex, 0.48092"

S18	:	"4 - Primary Motor Cortex, 0.43511"

D17	:	"6 - Pre-Motor and Supplementary Motor Cortex,"	1

S19	:	"6 - Pre-Motor and Supplementary Motor Cortex,"	1

D18	:	"6 - Pre-Motor and Supplementary Motor Cortex,"	1

S20	:	"1 - Primary Somatosensory Cortex, 0.045627"

S20	:	"3 - Primary Somatosensory Cortex, 0.39924"

S20	:	"4 - Primary Motor Cortex, 0.55513"

D19	:	"1 - Primary Somatosensory Cortex, 0.56537"

D19	:	"2 - Primary Somatosensory Cortex, 0.14134"

D19	:	"3 - Primary Somatosensory Cortex, 0.20141"

D19	:	"4 - Primary Motor Cortex, 0.063604"

D19	:	40 - Supramarginal gyrus part of Wernicke's ar	"ea, 0.028269"

S21	:	"2 - Primary Somatosensory Cortex, 0.12308"

S21	:	"22 - Superior Temporal Gyrus, 0.64615"

S21	:	"43 - Subcentral area, 0.22462"

S21	:	"48 - Retrosubicular area, 0.0061538"

D20	:	"20 - Inferior Temporal gyrus, 0.031546"

D20	:	"21 - Middle Temporal gyrus, 0.95899"

D20	:	"22 - Superior Temporal Gyrus, 0.0094637"

S22	:	"20 - Inferior Temporal gyrus, 0.66238"

S22	:	"21 - Middle Temporal gyrus, 0.23473"

S22	:	"37 - Fusiform gyrus, 0.10289"

D21	:	"21 - Middle Temporal gyrus, 0.053459"

D21	:	"22 - Superior Temporal Gyrus, 0.88679"

D21	:	"42 - Primary and Auditory Association Cortex,"	0.009434

D21	:	"48 - Retrosubicular area, 0.050314"

S23	:	40 - Supramarginal gyrus part of Wernicke's ar	"ea, 1"

D22	:	"1 - Primary Somatosensory Cortex, 0.0080645"

D22	:	"2 - Primary Somatosensory Cortex, 0.27823"

D22	:	"7 - Somatosensory Association Cortex, 0.10887"

D22	:	40 - Supramarginal gyrus part of Wernicke's ar	"ea, 0.60484"

S24	:	"1 - Primary Somatosensory Cortex, 0.14332"

S24	:	"3 - Primary Somatosensory Cortex, 0.2443"

S24	:	"4 - Primary Motor Cortex, 0.60586"

S24	:	"6 - Pre-Motor and Supplementary Motor Cortex,"	0.0065147

D23	:	"4 - Primary Motor Cortex, 1"

S25	:	"1 - Primary Somatosensory Cortex, 0.11881"

S25	:	"3 - Primary Somatosensory Cortex, 0.22772"

S25	:	"4 - Primary Motor Cortex, 0.62376"

S25	:	"6 - Pre-Motor and Supplementary Motor Cortex,"	0.029703

D24	:	"1 - Primary Somatosensory Cortex, 0.028112"

D24	:	"2 - Primary Somatosensory Cortex, 0.24498"

D24	:	"7 - Somatosensory Association Cortex, 0.14859"

D24	:	40 - Supramarginal gyrus part of Wernicke's ar	"ea, 0.57831"

S26	:	40 - Supramarginal gyrus part of Wernicke's ar	"ea, 1"

D25	:	"21 - Middle Temporal gyrus, 0.044728"

D25	:	"22 - Superior Temporal Gyrus, 0.87859"

D25	:	"48 - Retrosubicular area, 0.076677"

S27	:	"20 - Inferior Temporal gyrus, 0.65049"

S27	:	"21 - Middle Temporal gyrus, 0.29126"

S27	:	"37 - Fusiform gyrus, 0.058252"
