# Supplementary material for: Attenuated sensorimotor hemodynamic responses and altered cortical dynamics during passive proprioceptive-related stimulation after stroke: a multidimensional fNIRS study
Source: Front Neurol. 2026 Sep 16;17:1848276. doi: 10.3389/fneur.2026.1848276 (PMC13624074; doi:10.3389/fneur.2026.1848276)
Supplement: Supplementary file 2 [file Data_Sheet_4.DOCX]

1	1

2	2

3	3

4	4

5	5

6	6

7	7

8	8

9	9

10	10

11	11

12	12

13	13

14	14

15	15

16	16

17	17

18	18

19	19

20	20

21	21

22	22

23	23

24	24

25	30

26	26

27	27

28	28

29	29

30	25

31	31

32	32

33	33

34	34

35	35

36	36

37	37

38	47

39	46

40	45

41	44

42	43

43	42

44	41

45	40

46	39

47	38

48	58

49	57

50	56

51	55

52	54

53	53

54	52

55	51

56	50

57	49

58	48

59	68

60	67

61	66

62	65

63	64

64	63

65	62

66	61

67	60

68	59

69	79

70	78

71	77

72	76

73	75

74	74

75	73

76	72

77	71

78	70

79	69

80	89

81	88

82	87

83	86

84	85

85	84

86	83

87	82

88	81

89	80
