# Supplementary material for: Attenuated sensorimotor hemodynamic responses and altered cortical dynamics during passive proprioceptive-related stimulation after stroke: a multidimensional fNIRS study
Source: Front Neurol. 2026 Sep 16;17:1848276. doi: 10.3389/fneur.2026.1848276 (PMC13624074; doi:10.3389/fneur.2026.1848276)
Supplement: Supplementary file 3 [file Data_Sheet_5.DOCX]

**Supplementary Appendix 6
Detailed Statistical Analysis Plan and Parameter Reporting**

*For manuscript section 2.9 Statistical Analysis*

# 1. Streamlined 2.9 Statistical Analysis for the Main Text

Statistical analyses were performed using MATLAB. Continuous variables were assessed for distributional characteristics and homogeneity of variance, and were summarized as mean ± standard deviation or median (interquartile range), as appropriate. Between-group comparisons were performed using independent-samples t tests, Welch t tests, or Mann-Whitney U tests for continuous variables, and chi-square tests or Fisher exact tests for categorical variables. The main statistical analyses included ROI-level HbO temporal features, latent-state metrics, ROI-to-ROI directed connectivity, and recovery-phase complexity metrics. Return-to-baseline time was excluded from formal inference because it could not be reliably estimated in several regions. Multiple comparisons were controlled using the Benjamini-Hochberg false discovery rate procedure within predefined analytical families, including temporal-feature comparisons, latent-state metrics, phase-specific directed connectivity edges, and recovery-complexity metrics. Dominant pathways in the directed connectivity analysis were used descriptively to summarize the strongest stage-wise information-flow patterns and were not treated as independent inferential results. All tests were two-sided. Statistical significance was defined as P < 0.05 for analyses not involving multiple comparisons and as FDR-adjusted q < 0.05 for analyses involving multiple comparisons. Detailed module-specific statistical procedures and parameters are provided in Supplementary Appendix 6.

# 2. Purpose of This Supplementary Statistical Plan

This appendix provides the detailed statistical procedures that were shortened in the main text to improve readability. It clarifies the normality assessment, the strategy for false discovery rate correction, the handling of HbO temporal-feature p values, and the definition of dominant pathways in the directed connectivity analysis.

The appendix is intended to make the statistical workflow reproducible and to define which results are confirmatory, which are corrected for multiple comparisons, and which are descriptive or exploratory.

# 3. Summary of Statistical Issues Addressed

| **Reviewer concern** | **Clarification in the revision** | **Where addressed** |
| --- | --- | --- |
| Normality test not specified | Continuous variables are assessed using the Shapiro-Wilk test; homogeneity of variance is assessed using Levene's test. | Section 4.1 |
| Inconsistent FDR correction across analytical layers | Benjamini-Hochberg FDR correction is applied within predefined analytical families; raw p and FDR-adjusted q are reported where relevant. | Section 4.2 and Table 1 |
| Uncorrected HbO temporal-feature p values interpreted as significant | Significance is based on FDR-adjusted q < 0.05; uncorrected p < 0.05 with q >= 0.05 is described as nominal or exploratory. | Section 5.1 |
| Dominant pathways not defined | Dominant pathways are defined descriptively as the strongest group-level stage-wise PDC edges after excluding self-connections; they are not inferential results. | Section 5.4 |

# 4. General Statistical Principles

## 4.1 Distributional Assessment and Choice of Tests

All continuous variables were first evaluated for distributional characteristics using the Shapiro-Wilk test. Homogeneity of variance was assessed using Levene's test when parametric group comparison was considered.

Normally distributed continuous variables with homogeneous variances were compared using independent-samples t tests. When the normality assumption was acceptable but the equal-variance assumption was violated, Welch t tests were used. Non-normally distributed continuous variables were compared using Mann-Whitney U tests. Categorical variables were summarized as frequencies and percentages and compared using chi-square tests or Fisher exact tests, as appropriate.

All tests were two-sided. For analyses not involving multiple comparisons, P < 0.05 was considered statistically significant.

## 4.2 Multiple-Comparison Correction Strategy

For analytical modules involving multiple tests, the Benjamini-Hochberg false discovery rate procedure was applied within predefined analytical families. This approach controls the expected proportion of false discoveries while retaining sensitivity for exploratory neuroimaging analyses with multiple ROIs, states, or connection edges.

Both raw p values and FDR-adjusted q values should be reported for analyses involving multiple comparisons. Statistical significance for these analyses is determined by q < 0.05. Results with uncorrected p < 0.05 but q >= 0.05 should be described as nominal, trend-level, or exploratory findings rather than statistically significant findings.

**Table 1. Predefined analytical families for FDR correction**

| **Analytical module** | **Tests included in the FDR family** | **Decision rule** |
| --- | --- | --- |
| HbO temporal-feature analysis | Group comparisons across predefined ROIs and temporal metrics: AUC, onset latency, peak amplitude, post-stimulus residual activation, recovery slope, and time to peak. | q < 0.05 indicates statistical significance. |
| Latent-state analysis | State-wise group comparisons of fractional occupancy and mean dwell time. Transition matrices are mainly descriptive unless a separate hypothesis-driven test is prespecified. | q < 0.05 for state-wise metrics. |
| Directed connectivity analysis | ROI-to-ROI PDC edge-wise group comparisons within each task phase: pre-stimulation, stimulation, and recovery. | q < 0.05 within each phase-specific edge family. |
| Recovery-phase complexity, global level | Global SampEn, MeanMSE, RR, DET, and Lmean. | q < 0.05 across the set of global metrics. |
| Recovery-phase complexity, ROI level | ROI-wise group comparisons within each specific complexity metric. | q < 0.05 within each metric family across ROIs. |

# 5. Module-Specific Statistical Procedures

## 5.1 HbO Temporal-Feature Analysis

ROI-level HbO temporal features were compared between groups at the level of predefined regions of interest. The analyzed metrics were area under the curve, onset latency, peak amplitude, post-stimulus residual activation, recovery slope, and time to peak. Return-to-baseline time was excluded from formal inference because it could not be reliably estimated in several regions.

The temporal-feature analysis was performed on non-normalized block-averaged HbO curves so that amplitude-related metrics retained their original relative HbO amplitude information. Group comparisons were conducted using distribution-appropriate independent-samples tests. Multiple comparisons across ROI-by-feature tests were corrected using the Benjamini-Hochberg FDR procedure.

For reporting Figure 2 and the corresponding results, uncorrected p values may be shown for transparency, but statistical significance should be based on FDR-adjusted q values. If p < 0.05 but q >= 0.05, the result should be described as a nominal or exploratory group difference, not as a statistically significant difference.

## 5.2 Latent-State Analysis

The optimal number of latent states was determined before group comparison using model comparison. Candidate models should be reported with their corresponding AIC and BIC values. The model with the lowest BIC was selected as the main solution.

Subject-level state metrics, including fractional occupancy and mean dwell time for each state, were compared between groups using distribution-appropriate independent-samples tests. Multiple state-wise comparisons were corrected using the Benjamini-Hochberg FDR procedure within each metric family.

The transition probability matrix was used primarily to describe organizational differences in state transitions between groups. Individual transition elements were not treated as separate confirmatory statistical tests unless explicitly prespecified.

## 5.3 Directed Connectivity Analysis

Partial directed coherence was calculated separately for the pre-stimulation, stimulation, and recovery phases. For inferential analysis, between-group comparisons were performed for each directed ROI-to-ROI edge within each phase. Because the number of directed edges was large, Benjamini-Hochberg FDR correction was applied separately within each phase-specific edge family.

The directed connectivity results should be interpreted as phase-specific directed coupling patterns. Edges are considered statistically significant only when the corresponding FDR-adjusted q value is < 0.05.

## 5.4 Definition of Dominant Pathways

Dominant pathways were defined descriptively, not inferentially. For each group and each task phase, subject-level PDC matrices were averaged within phase and then averaged across subjects to obtain a group-level stage-wise mean PDC matrix. Self-connections were excluded. Directed ROI-to-ROI edges were ranked in descending order according to mean PDC strength.

The strongest ranked edges were displayed to summarize the major information-flow configuration and its migration across task phases. These dominant pathways were not treated as statistically significant edges unless supported by the corresponding edge-wise group comparison and FDR correction. Therefore, wording such as "dominant pathway migration" or "strongest directed coupling configuration" is preferred over causal or confirmatory phrasing.

## 5.5 Recovery-Phase Complexity Analysis

Recovery-phase complexity was analyzed at both global and ROI levels. Global metrics included SampEn, MeanMSE, RR, DET, and Lmean. Distribution-appropriate independent-samples tests were used for between-group comparisons, with FDR correction applied across the global metric family.

For ROI-level complexity analysis, each complexity metric was compared between groups across all ROIs. FDR correction was applied within each metric family across ROIs. The distinction between global-level and ROI-level correction families should be clearly maintained in the results.

# 6. Recommended Reporting Wording

Use the following wording to maintain consistency between the statistical methods, results, and figure legends:

- If a result survives FDR correction: "The group difference remained significant after FDR correction (p = ..., q = ...)."
- If only the uncorrected p value is < 0.05: "The group difference reached nominal significance before correction (p = ...), but did not survive FDR correction (q = ...)."
- For Figure 2 legend: "Raw p values and FDR-adjusted q values are reported. Statistical significance was determined according to FDR-adjusted q < 0.05."
- For directed connectivity: "Dominant pathways were defined as the strongest directed edges ranked by group-level mean PDC strength and were used for descriptive visualization only."

# 7. Key Methodological References

1. Benjamini Y, Hochberg Y. Controlling the false discovery rate: a practical and powerful approach to multiple testing. Journal of the Royal Statistical Society: Series B. 1995;57(1):289-300.
2. Shapiro SS, Wilk MB. An analysis of variance test for normality. Biometrika. 1965;52(3/4):591-611.
3. Levene H. Robust tests for equality of variances. In: Olkin I, Ghurye SG, Hoeffding W, Madow WG, Mann HB, editors. Contributions to Probability and Statistics. Stanford University Press; 1960. p. 278-292.
4. Baccala LA, Sameshima K. Partial directed coherence: a new concept in neural structure determination. Biological Cybernetics. 2001;84(6):463-474.
