# Supplementary material for: Attenuated sensorimotor hemodynamic responses and altered cortical dynamics during passive proprioceptive-related stimulation after stroke: a multidimensional fNIRS study
Source: Front Neurol. 2026 Sep 16;17:1848276. doi: 10.3389/fneur.2026.1848276 (PMC13624074; doi:10.3389/fneur.2026.1848276)
Supplement: Supplementary file 4 [file Data_Sheet_6.DOCX]

II. Figure 2: HbO Temporal Characteristics

------------------------------------------------------------

A. Raw p-values for each ROI and metric

ROI	| AUC	| Onset latency | Peak amplitude | Post-stimulus residual activation | Recovery slope | Time-to-peak

------|-------|---------------|----------------|-----------------------------------|----------------|-------------

LPFC | 0.805 | 0.734	| 0.667	| 0.098	| 0.240	| 0.467

LSMC | 0.023 | 0.482	| 0.003	| 0.961	| 0.018	| 0.231

RPFC | 0.540 | 0.380	| 0.838	| 0.949	| 0.773	| 0.077

RSMC | 0.184 | 0.340	| 0.371	| 0.285	| 0.051	| 0.893

B. Key results reported in the main text

1. AUC

ROI: LSMC

Direction: Stroke < Health

Test method: Wilcoxon rank-sum test

Raw p-value: 0.023

2. Peak amplitude

ROI: LSMC

Direction: Stroke < Health

Test method: Wilcoxon rank-sum test

Raw p-value: 0.003

3. Recovery slope

ROI: LSMC

Direction: The slope in the stroke group was less negative (closer to zero).

Interpretation: The post-peak decline of HbO was more gradual, indicating slower recovery.

Test method: Wilcoxon rank-sum test

Raw p-value: 0.018

Note:

This text file contains only the test methods and p-values; it does not include descriptive statistics, rank-sum statistics, or effect sizes for the two groups.

If effect sizes are to be fully reported in Attachment 8, additional calculations of rank-biserial correlation or Cliff's delta should be performed based on the raw individual-level data. III. Figure 3: Latent State Metrics

------------------------------------------------------------

Optimal number of states: K = 6

A. Fractional occupancy (FO)

State 1

Health = 0.0922

Stroke = 0.0351

Direction: Health > Stroke

State 3

Health = 0.1245

Stroke = 0.1887

Direction: Stroke > Health

State 5

Health = 0.1315

Stroke = 0.0585

Direction: Health > Stroke

State 6

Health = 0.0944

Stroke = 0.2055

Direction: Stroke > Health

B. Mean dwell time (MDT)

State 2

Health = 6.0600 s

Stroke = 7.5170 s

Direction: Stroke > Health

State 3

Health = 4.7760 s

Stroke = 8.2612 s

Direction: Stroke > Health

Note:

The current report provides group means for FO and MDT across states but does not fully report the exact p-values,

test statistics, and effect sizes for between-group comparisons. Upon finalization of Attachment 8, the following

details should be supplemented from the original analysis results:

statistical test methods, test statistics, raw p-values, FDR-corrected q-values, effect sizes, and 95% confidence intervals.

IV. Figure 4: PDC Directed Connectivity

------------------------------------------------------------

Twelve predefined key pathways were analyzed for each stage. Summary of statistical results by phase:

Pre-stimulation: 1 pathway with raw P < 0.05; 0 significant after FDR correction

Stimulation: 3 pathways with raw P < 0.05; 0 significant after FDR correction

Recovery: 2 pathways with raw P < 0.05; 0 significant after FDR correction

Key nominally significant pathways:

1. Pre-stimulation

Pathway: L-S -> L-dlPFC

Health = 0.137991 ± 0.117773

Stroke = 0.227302 ± 0.180381

Test: Wilcoxon rank-sum test

Raw P = 0.040

FDR q = 0.296

Direction: Stroke > Health

2. Stimulation

Pathway: L-S -> L-dmPFC

Health = 0.148775 ± 0.083385

Stroke = 0.117188 ± 0.082617

Test: Wilcoxon rank-sum test

Raw P = 0.046

FDR q = 0.296

Direction: Health > Stroke

3. Stimulation

Pathway: R-S -> R-PMC

Health = 0.252134 ± 0.121951

Stroke = 0.179012 ± 0.137631

Test: Wilcoxon rank-sum test

Raw P = 0.007

FDR q = 0.252

Direction: Health > Stroke

4. Stimulation

Pathway: R-S -> R-dmPFC

Health = 0.155169 ± 0.069098

Stroke = 0.121750 ± 0.077028

Test: Wilcoxon rank-sum test

Raw P = 0.038

FDR q = 0.296 Direction: Health > Stroke

5. Recovery

Pathway: R-S -> R-PMC

Health = 0.208376 ± 0.091294

Stroke = 0.168492 ± 0.109494

Test: Wilcoxon rank-sum test

Raw P = 0.049

FDR q = 0.296

Direction: Health > Stroke

6. Recovery

Pathway: R-S -> R-dmPFC

Health = 0.173278 ± 0.091916

Stroke = 0.123103 ± 0.080654

Test: Two-sample t-test

Raw P = 0.026

FDR q = 0.296

Direction: Health > Stroke

Conclusion:

No predefined directed pathway survived FDR correction in any phase.

Note:

The edges listed above are merely nominally significant or exploratory results; they cannot be described as showing significant differences after FDR correction. V. Figure 5: Complexity during the recovery phase

------------------------------------------------------------

1. SampEn

Health = 0.101057 ± 0.030632

Stroke = 0.087794 ± 0.025539

Test: Two-sample t-test

Raw P = 0.070

FDR q = 0.070

Direction: Health > Stroke

Significant: No

2. MeanMSE

Health = 0.267253 ± 0.059487

Stroke = 0.235621 ± 0.061675

Test: Two-sample t-test

Raw P = 0.044

FDR q = 0.055

Direction: Health > Stroke

Significant: No

3. RR

Health = 0.075052 ± 0.016321

Stroke = 0.081441 ± 0.014996

Test: Wilcoxon rank-sum test

Raw P = 0.023

FDR q = 0.049

Direction: Stroke > Health

Significant: Yes

4. DET

Health = 0.948835 ± 0.025671

Stroke = 0.961876 ± 0.017631

Test: Wilcoxon rank-sum test

Raw P = 0.022

FDR q = 0.049

Direction: Stroke > Health

Significant: Yes

5. Lmean

Health = 8.381780 ± 2.537082

Stroke = 10.321313 ± 4.920711

Test: Wilcoxon rank-sum test

Raw P = 0.030

FDR q = 0.049

Direction: Stroke > Health Significant: Yes

VI. Attachment 8: Recommended standardized table headers

------------------------------------------------------------

Analysis module

Outcome

ROI / State / Pathway

Health

Stroke

Statistical test

Test statistic

Raw P

FDR-adjusted q

Effect size

95% CI
